# Proteome profiling of early gestational plasma reveals novel biomarkers of congenital heart disease

**DOI:** 10.15252/emmm.202317745

**Published:** 2023-10-16

**Authors:** Ya‐Nan Yin, Li Cao, Jie Wang, Yu‐Ling Chen, Hai‐Ou Yang, Su‐Bei Tan, Ke Cai, Zhe‐Qi Chen, Jie Xiang, Yuan‐Xin Yang, Hao‐Ran Geng, Ze‐Yu Zhou, An‐Na Shen, Xiang‐Yu Zhou, Yan Shi, Rui Zhao, Kun Sun, Chen Ding, Jian‐Yuan Zhao

**Affiliations:** ^1^ Institute for Developmental and Regenerative Cardiovascular Medicine, MOE‐Shanghai Key Laboratory of Children's Environmental Health, Xinhua Hospital Shanghai Jiao Tong University School of Medicine Shanghai China; ^2^ State Key Laboratory of Genetic Engineering and Collaborative Innovation Center for Genetics and Development, School of Life Sciences Institutes of Biomedical Sciences, Human Phenome Institute, Zhongshan Hospital, Fudan University Shanghai China; ^3^ National Health Commission (NHC) Key Laboratory of Neonatal Diseases, School of Life Sciences Obstetrics and Gynecology Hospital of Fudan University, Children's Hospital of Fudan University, Fudan University Shanghai China; ^4^ International Peace Maternity and Child Health Hospital of China Welfare Institute Shanghai Jiao Tong University School of Medicine Shanghai China; ^5^ International Human Phenome Institutes (Shanghai) Shanghai China; ^6^ School of Basic Medical Sciences Zhengzhou University Zhengzhou China

**Keywords:** congenital heart disease, plasma, proteomics gestational, Biomarkers, Cardiovascular System, Development

## Abstract

Prenatal diagnosis of congenital heart disease (CHD) relies primarily on fetal echocardiography conducted at mid‐gestational age—the sensitivity of which varies among centers and practitioners. An objective method for early diagnosis is needed. Here, we conducted a case–control study recruiting 103 pregnant women with healthy offspring and 104 cases with CHD offspring, including VSD (42/104), ASD (20/104), and other CHD phenotypes. Plasma was collected during the first trimester and proteomic analysis was performed. Principal component analysis revealed considerable differences between the controls and the CHDs. Among the significantly altered proteins, 25 upregulated proteins in CHDs were enriched in amino acid metabolism, extracellular matrix receptor, and actin skeleton regulation, whereas 49 downregulated proteins were enriched in carbohydrate metabolism, cardiac muscle contraction, and cardiomyopathy. The machine learning model reached an area under the curve of 0.964 and was highly accurate in recognizing CHDs. This study provides a highly valuable proteomics resource to better recognize the cause of CHD and has developed a reliable objective method for the early recognition of CHD, facilitating early intervention and better prognosis.

## Graphical abstract

Mass spectrometry‐based proteomics for plasma proteome profiling was performed on early gestational pregnant women with or without congenital heart disease (CHD) offspring. In‐depth analysis revealed a potential pathogenic mechanism and identified a set of biomarkers in early gestational plasma predicting fetal CHD.

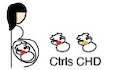

**The paper explained**

**Problem**

Congenital heart disease (CHD) is the most common congenital malformation in newborns, which brings a huge burden to the patients, their families, and society. Currently, there is still a lack of reliable biomarkers for prenatal diagnosis of CHD in early pregnancy.

**Results**

We performed proteomic analysis of 103 pregnant women with CHD offspring and 103 controls with healthy offspring to determine the biomarkers of CHD in maternal plasma during early pregnancy (10–12 weeks gestation) and establish a molecular prenatal diagnostic method. The results revealed that the combination of nine proteins was highly accurate in identifying CHD cases.

**Impact**

The plasma proteome during pregnancy provides a better understanding of the pathophysiology of congenital heart defects, including changes in plasma protein levels and the correlation between protein and clinical parameters. In addition, candidate biomarkers for early diagnosis of CHD have been developed.

## Introduction

Congenital heart disease (CHD) is the most common type of birth defect globally (van der Linde *et al*, 2011; Zhang *et al*, 2021a). Owing to its high incidence and fatality rate, the prompt detection of CHD in early pregnancy is imperative for prevention and treatment (Botto, 2000). The earlier the diagnosis of CHD, the better the prognosis (Sadeck Ldos *et al*, 1997). Thus, the prenatal detection of fetal CHD is necessary to minimize mortality and improve the prognosis of fetuses with CHD.

Currently, even with the advent of fetal echocardiography as a screening tool for CHD, cardiac abnormalities are still overlooked during routine prenatal care, with dismal detection rates ranging from 6 to 35% (Sharland & Allan, 1992; Garne *et al*, 2001; Jaeggi *et al*, 2001). In addition, ultrasound examination results vary among centers due to a lack of standardization (Friedberg *et al*, 2009). The accuracy of prenatal sonographic investigations to detect CHD is influenced by many factors, such as the experience of operators, the quality of the ultrasound equipment, lesion type, departmental policies, and guidelines (Stumpflen *et al*, 1996; Fernandez *et al*, 1998; Stoll *et al*, 1998; Isaksen *et al*, 1999; Allan, 2000). This also lowers the CHD detection rate in underdeveloped regions than that in developed regions. Therefore, the development of novel methods for the early diagnosis of CHD has become crucial for the prevention and treatment of birth defects.

In addition to imaging methods (Wang *et al*, 2021), genetic and biochemical methods may be used for the early detection of CHD. Genetic variants in both the pregnant mother and offspring predict the risk of CHD in the offspring. Various single nucleotide polymorphisms in folate metabolism genes are associated with the risk of CHD (Zhao *et al*, 2012, 2013, 2014; Wang *et al*, 2014, 2017). However, because these variants commonly occur in the population, their applicability in predicting the risk of CHD in offspring is unsatisfactory. Recently, maternal biomarkers have been found to be associated with fetal CHD *in utero*, including increased levels of free beta‐human chorionic gonadotropin (β‐hCG) and branched‐chain amino acids (Zhang *et al*, 2022), and decreased levels of pregnancy‐associated plasma protein‐A (PAPP‐A) in the first trimester (Michailidis & Economides, 2001; Souter *et al*, 2002; Makrydimas *et al*, 2003; Jelliffe‐Pawlowski *et al*, 2008). In addition, the altered profile of blood microRNAs and lncRNAs has also been explored as a novel biomarker for the prenatal diagnosis of fetal CHD (Zhu *et al*, 2013; Gu *et al*, 2016). However, the current progress in identifying biomarkers still cannot meet the urgent need for improved biomarkers for the early diagnosis of CHD.

Irrespective of the cause of CHD, such as genetic, nutritional, or environmental, proteins serve as the molecular machines underlying cardiovascular development. Alterations in the activity of disease‐related proteins can lead to disease occurrence. Alternatively, protein changes may also be a consequence of CHD because structural and functional fetal cardiovascular system development defects might reflect in the protein composition of maternal peripheral blood. Regardless of whether they are the causes or consequences of CHD, these protein changes in cardiovascular cells during the expected phase of fetal heart development may lead to subsequent changes in protein levels in the maternal blood, which can be detected in early pregnancy and provide a new opportunity for the early diagnosis of CHD.

Blood has always been a promising source of clinical and biological research and may contain any type of protein found in human cells (Chen *et al*, 2023b). Proteins in the blood not only reflect physiological and pathological conditions but also include biomarkers of disease and therapeutic efficacy (Ku *et al*, 2023). This study aimed to perform an unbiased proteomics analysis of plasma proteins in pregnant women in the first trimester with and without CHD fetuses to comprehensively identify novel diagnostic biomarkers. A set of reliable protein biomarkers, instead of a single marker, could also enable the development of highly specific tests for diagnosis in early pregnancy and may provide new insights into the mechanisms underlying CHD.

## Results

### Proteomic characterization of maternal plasma

**Figure 1:**
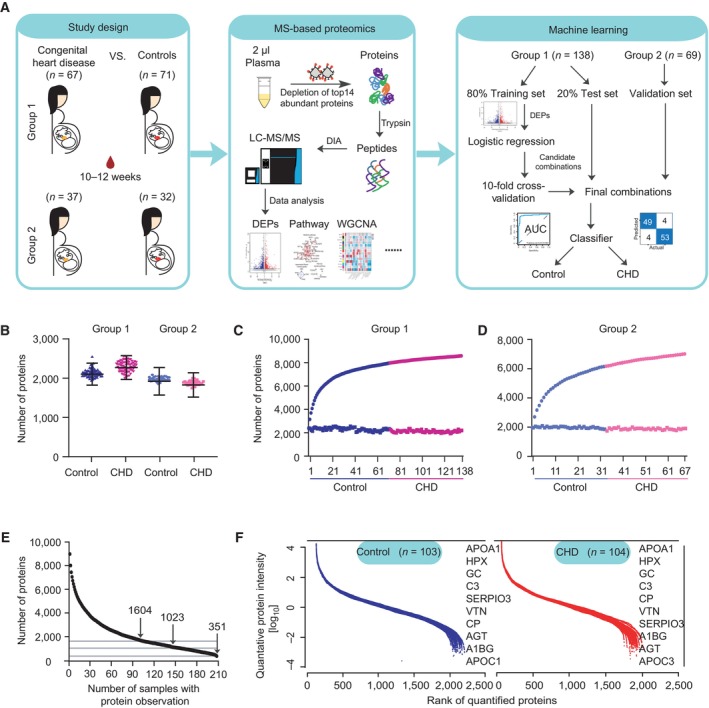
Research overview and proteomic characterization of maternal plasma Overview of the investigated groups and a schematic diagram of the proteomic analysis. Maternal blood samples were collected at 10–12 weeks of gestation. The total number of participants in each group and the basic procedures for proteomic analysis are described.Proteins were quantified using a 1% false discovery rate (FDR) cutoff. Values are reported as mean ± standard deviation (SD). *n* = 71 (control in group 1), 67 (CHD offspring in group 1), 32 (control in group 2), and 37 (CHD offspring in group 2) as biological replicates.Cumulative number of proteins identified (the left panel shows the results for control and the right panel shows the results for CHD) in group 1. The number of proteins in the dataset (*Y*‐axis) was plotted against the number of samples (*X*‐axis).Cumulative number of proteins identified (the left panel shows the results for the control and the right panel shows the results for CHD) in group 2. The number of proteins in the dataset (*Y*‐axis) was plotted against the number of samples (*X*‐axis).Data completeness curve. The number of proteins in the dataset (*Y*‐axis) is plotted against the minimum number of samples in which the proteins were quantified (*X*‐axis). Arrows indicate data completeness values of 50, 75, and 100%.The protein abundance distributions in the CHD group (red) and the control group (blue) are plotted. The top 10 most abundant proteins are indicated in the box. Source data are available online for this figure.

To map plasma proteome changes during early pregnancy between individuals with CHD offspring and healthy offspring, we analyzed two independent case–control groups, recruiting 206 individuals in total. These included 67 patients with CHD offspring and 71 controls in group 1 recruited from the Obstetrics & Gynecology Hospital of Fudan University, and 37 cases and 32 controls in group 2 recruited from the International Peace Maternity and Child Health Hospital of the China Welfare Institute, amounting to 103 controls and 104 cases, with the most frequent phenotypes VSD (42/104) and ASD (20/104) (Fig 1A, Dataset EV1).

**Figure EV1:**
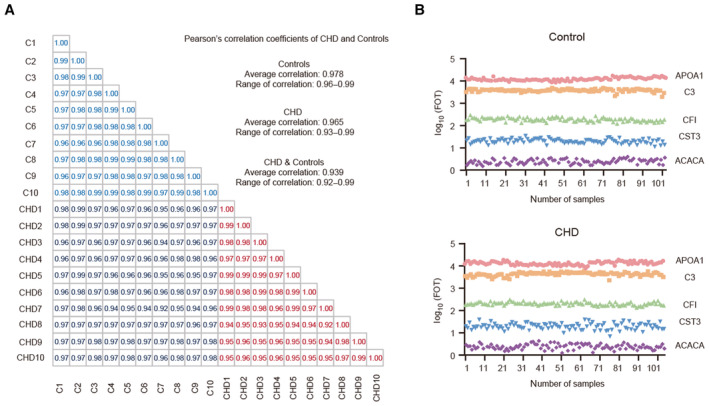
Reproducibility of plasma data Pearson's correlation coefficients for replicate proteome profiling of 20 plasma samples (10 CHD and 10 healthy control samples).Reproducibility of the fraction of total (FOT) of six proteins in 207 samples. FOT was defined as the iBAQ of a protein divided by the total iBAQ of all identified proteins within a sample.

The experimental workflow is shown in Fig 1A. LC–MS/MS analysis was conducted based on the data‐independent acquisition (DIA) method in all the samples from both groups, and all plasma proteomics data were analyzed. Applying this robust workflow, we quantified an average of 2,220 (group 1) and 1,926 (group 2) proteins per plasma sample (Fig 1B; Datasets EV2 and EV3). There were no outliers, and all samples could be used for further analysis (Fig 1B). Furthermore, we identified 8,624 and 7,049 proteins in groups 1 and 2, respectively (Fig 1C and D). The number of proteins gradually plateaued as the number of samples increased, indicating deep coverage and good stability of protein detection. Fewer proteins were identified in the plasma samples from group 2 than those from group 1, which may be attributed to the batch effect. Nevertheless, we achieved a single‐shot high‐throughput workflow with deep proteomic coverage from 2 μl of plasma samples (Fig 1E). Among the data acquired by DIA, there were 351 proteins with 100% completeness, 1,023 proteins with 75% completeness, and 1,604 proteins with 50% completeness (Fig 1E). In all the samples, the quantitative protein intensities in the control and CHD groups spanned 8 orders of magnitude, and the top 10 highly abundant proteins contributed to 40 and 39%, respectively, of all plasma protein abundance in our datasets (Fig 1F). In addition, the reproducibility of the proteomic data was assessed by analyzing the abundance of correlated proteins across the whole measuring range. Twenty samples (10 CHD and 10 control) from 207 samples were randomly selected for the analysis. The results showed that the average correlation value of quantified protein signals between individual replicates in the healthy control group was 0.978, with a range of 0.96–0.99. Similarly, the average correlation value in the CHD group was 0.965, with a range of 0.93–0.99. The average correlation value between CHD and healthy control was 0.939 (Fig EV1A). Furthermore, to illustrate the good reproducibility of data within each case, we picked five well‐characterized plasma proteins and found that the quantification was highly reproducible in 103 healthy control and 104 CHD cases (Fig EV1B).

### Detection of CHD‐related proteome alterations in plasma

**Figure 2:**
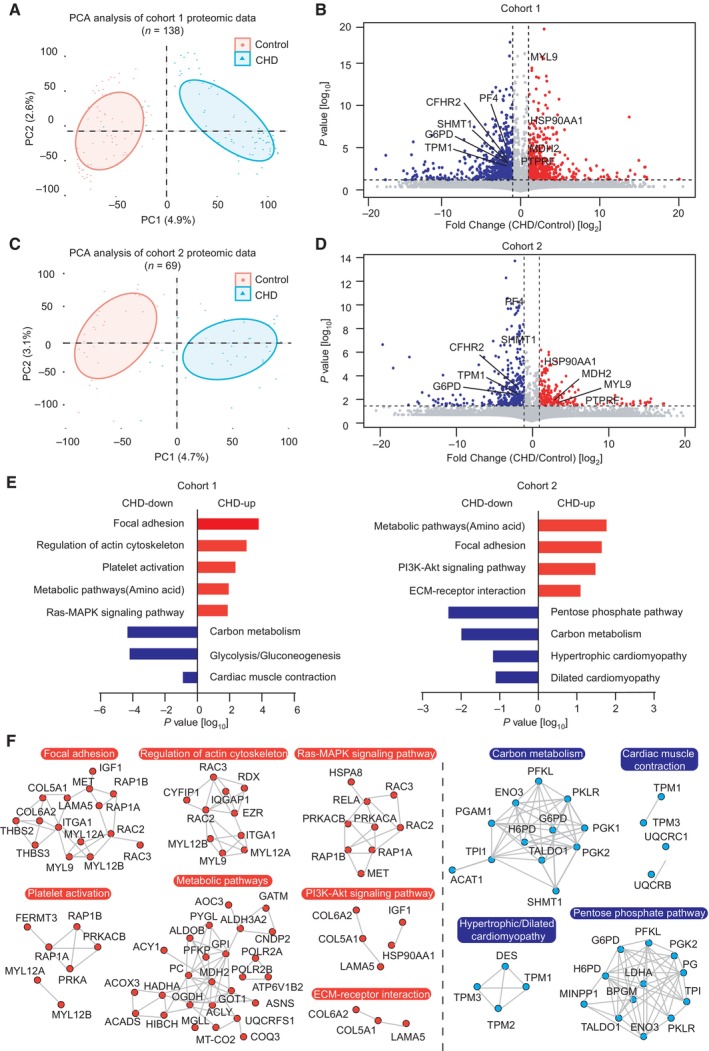
Differences in CHD versus control plasma proteome in the two groups Principal component analysis (PCA) of proteins in plasma samples from group 1. Control participants are represented in red and CHD in blue.Relationship between fold‐change values for CHD/control samples and statistical significance for group 1. Red indicates upregulated proteins, blue indicates downregulated proteins, and proteins above the gray dotted line are statistically significant (*P* < 0.05).PCA of plasma proteins from group 2. Control participants are represented in red, and CHD in blue.Relationship between fold‐change values for CHD/control samples and statistical significance for group 2. Red indicates upregulated proteins, blue indicates downregulated proteins, and proteins above the gray dotted line are statistically significant (*P* < 0.05).Gene ontology (GO) annotations with upregulated or downregulated proteins (*P* < 0.05).Protein interaction analysis of enriched pathways in the two groups is shown in (E). Red indicates interactions between upregulated proteins, whereas blue indicates interactions between downregulated proteins. Source data are available online for this figure.

**Figure EV2:**
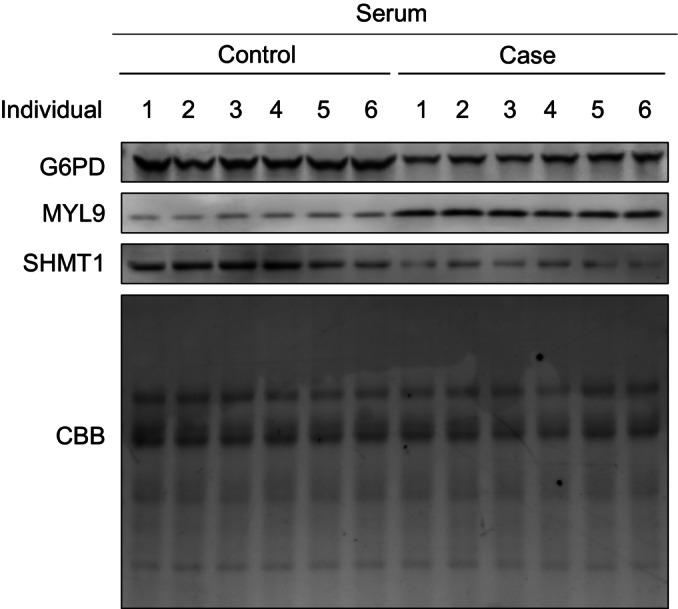
Protein levels in serum from pregnant women with CHD or normal offspring Protein levels of G6PD, MYL9, and SHMT1 in serum from both control and case groups were detected by western blotting. CBB, Coomass.

Principal component analysis (PCA) showed a clear difference between cases and controls in both groups, indicating that during early pregnancy, pregnant women with CHD fetuses exhibit plasma proteomic characteristics different from those in pregnant women carrying healthy fetuses (Fig 2A and C). Furthermore, we identified the individual molecular features associated with CHD in both groups. Group 1 revealed 397 differentially expressed proteins (DEPs) between the control and CHD groups. Among these, 184 significantly upregulated and 213 downregulated proteins (Student's *t*‐test, *P* < 0.05, and fold change > 2 or < 0.5) were detected in the CHD group (Fig 2B, Dataset EV4). In group 2, 225 DEPs were identified between the control and CHD groups. Among these, 80 proteins were significantly upregulated and 145 proteins were significantly downregulated (Student's *t*‐test, *P* < 0.05, and fold change > 2 or < 0.5) in the CHD group (Fig 2D, Dataset EV5). These DEPs included several previously identified plasma CHD markers, such as neuropilin‐2, ATP‐citrate synthase (ACLY), protein S100‐A7 (S100A7), myosin regulatory light polypeptide 9 (MYL9), glucose‐6‐phosphate 1‐dehydrogenase (G6PD), serine hydroxymethyltransferase (SHMT1), coactosin‐like protein (COTL1), and plasminogen activator inhibitor 1 (SERPINE1) (Jain *et al*, 2004; Nembhard *et al*, 2017; Song *et al*, 2017; Ference *et al*, 2019; Xiong *et al*, 2019; Zhang *et al*, 2019; Tan *et al*, 2020; Luo *et al*, 2022). In addition, we identified other significantly changed proteins, such as deoxynucleoside triphosphate triphosphohydrolase 1 (SAMHD1) and secreted protein acidic and rich in cysteine (SPARC). We also validated the proteomics results of protein abundance using western blotting and confirmed the decreased expression of G6PD and SHMT1, and the increased expression of MYL9 in plasma samples from pregnant women with CHD offspring (Fig EV2).

To further analyze the profile of altered proteins in maternal plasma, we annotated significant DEPs in both groups using gene ontology (GO) term analysis and identified the biological processes influenced by fetal CHD. In both groups, 264 significantly upregulated proteins in the CHD groups were mainly enriched in amino acid metabolism, extracellular matrix (ECM) receptor, actin skeleton regulation, Ras‐MAPK signaling pathway, and PI3K‐Akt signaling pathway. Conversely, 358 significantly downregulated proteins were strongly associated with carbohydrate metabolism, cardiac muscle contraction, and cardiomyopathy (Fig 2E). In addition, to understand the molecular pathway relationships in the various up‐ and downregulated proteins, protein–protein interactions (PPI) network analysis was performed on the altered proteins, revealing key molecules in each pathway (Fig 2F, Tables EV1 and EV2). These results revealed that the maternal plasma of pregnant women with CHD fetuses expressed a large number of proteins related to embryonic organ development, which was significantly different from that with healthy fetuses, and these significantly altered proteins might potentially serve as biomarkers of disease.

### Replication of CHD‐associated proteins in the two groups

**Figure 3:**
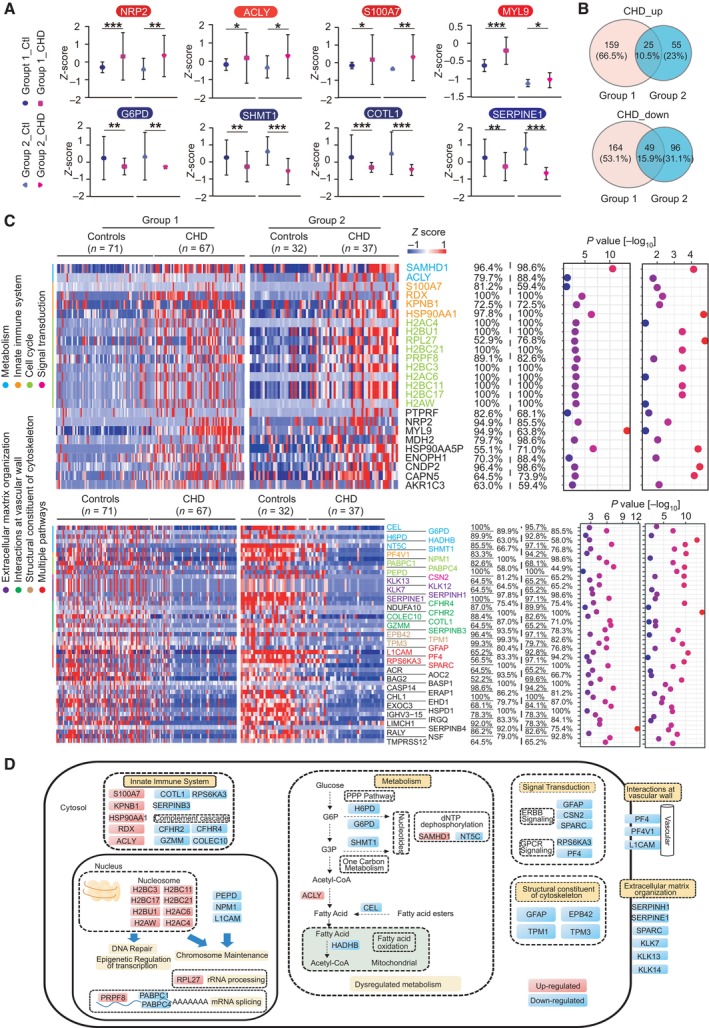
Plasma proteome alterations in the two groups Expression levels of previously reported heart disease‐related proteins. ***P < 0.001; **P < 0.01; *P < 0.05; *P*‐values from unpaired *t*‐test are shown. Lines indicate mean and SD. *n* = 71 (control in group 1), 67 (CHD offspring in group 1), 32 (control in group 2), and 37 (CHD offspring in group 2) as biological replicates.Venn diagram of up‐ and downregulated proteins.Expression levels and pathway enrichment of identified proteins in both groups. The heatmaps indicate the expression levels, frequencies, and *P*‐values for 25 upregulated proteins in the two groups (upper panel) and 49 downregulated proteins in the two groups (lower panel).Pathway patterns of differentially expressed proteins in the two groups. Source data are available online for this figure.

The expression levels of eight known CHD‐related proteins, including heat shock protein HSP 90‐alpha (HSP90AA1) (Sevim Bayrak *et al*, 2020; Xiao *et al*, 2020), malate dehydrogenase (MDH2) (Liu *et al*, 2014), MYL9 (Xiong *et al*, 2019), ACLY (Ference *et al*, 2019), N‐ethylmaleimide sensitive factor (NSF) (Mei *et al*, 2020), α‐tropomyosin gene (TPM) (Hirono *et al*, 2020; Zhang *et al*, 2020), SERPINE1 (Song *et al*, 2017), and complement factor H related 2 (CFHR2) (Zhang *et al*, 2016) were altered consistently in both groups. The protein expression levels of HSP90AA1, MDH2, MYL9, and ACLY were higher in the CHD group than those in the control group. In contrast, the protein expression levels of NSF, TPM1, SERPINE1, and CFHR2 were lower in the CHD group than those in the control group (Fig 3A). These results suggested that maternal plasma‐expressed proteins were associated with heart development.

Furthermore, changes in the levels of 74 proteins between the CHD and control plasma were consistent between group 1 and group 2. In total, 25 proteins were significantly upregulated and 49 proteins were significantly downregulated in both groups (Fig 3B; Table EV3). We further analyzed these 74 DEPs, which revealed that the 25 upregulated proteins were mainly involved in metabolism, innate immune response, and cell cycle pathways, whereas the 49 downregulated proteins were mainly involved in processes such as glucose metabolism, lipid metabolism, and vascular interaction. These pathways may be essential for fetal heart development (Fig 3C and D).

### Construction and verification of a CHD protein co‐expression network

**Figure 4:**
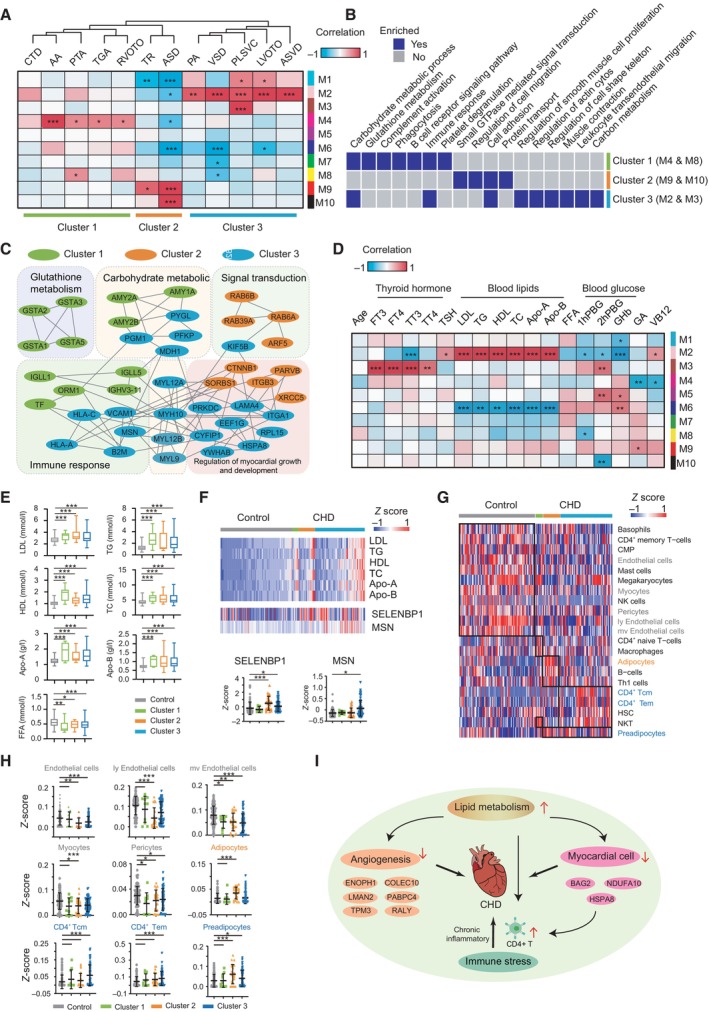
Relationship between CHD plasma proteome and clinical phenotypes and indicators Weighted gene co‐expression network analysis (WGCNA) of 207 plasma samples showed that 12 pathological features of CHD could be integrated into three clusters according to the correlations between module proteins. The strengths of the positive (red) and negative (blue) correlations are illustrated in the two‐color heatmap. Pearson correlation coefficients and *P*‐values were calculated by WGCNA package. ***P < 0.001; **P < 0.01; *P < 0.05.Gene ontology (GO) analysis of proteins in each cluster.Protein–protein interaction analysis of three major pathological features.Analysis of changes in different CHD protein networks and their correlation with clinical indicators.Expression levels of blood lipids in the controls and CHDs. ***P < 0.001; **P < 0.01; *P < 0.05; *P*‐values from unpaired *t*‐test are shown. Whiskers mark minimum or maximum values. *n* = 103 (Control), 10 (Cluster 1), 23 (Cluster 2), and 71 (Cluster 3) as biological replicates.Correlations of protein expression with clinical indicators (blood lipids). ***P < 0.001; *P < 0.05; *P*‐values from unpaired *t*‐test are shown. Lines indicate mean and SD. *n* = 103 (Control), 10 (Cluster 1), 23 (Cluster 2), and 71 (Cluster 3) as biological replicates.Heatmap of cluster types of specific immune cells in the controls and CHDs.Scatter plot illustrating the xCell scores for specific cell types in the control and CHDs groups. ***P < 0.001; **P < 0.01; *P < 0.05; *P*‐values from unpaired *t*‐test are shown. Lines indicate mean and SD. *n* = 103 (Control), 10 (Cluster 1), 23 (Cluster 2), and 71 (Cluster 3) as biological replicates.Blood lipids and the potential pathogenesis of CHD. Source data are available online for this figure.

To identify the potential drivers of CHD pathology, we included proteins with less than 30% missing values in subsequent analyses, and approximately 2,280 proteins were selected to generate a protein co‐expression network using weighted gene co‐expression network analysis (WGCNA). The co‐expression network consisted of 10 protein modules (M1‐M10), the size of which ranged from 20 to 492 proteins (Fig 4A; Dataset EV6). Subsequently, hierarchical clustering analysis of 12 clinicopathological phenotypes was classified into the following three phenotype clusters: cluster 1, including Tetralogy of Fallot (TOF), aortic stenosis (AS), persistent truncus arteriosus (PTA), transposition of the great arteries (TGA), and right ventricular outflow tract obstruction (RVOTO); cluster 2, including tricuspid regurgitation (TR) and atrial septal defect (ASD); and cluster 3, including pulmonary stenosis (PS), ventricular septal defect (VSD), persistent left superior vena cava (PLSVC), left ventricular outflow tract obstruction (LVOTO), and atrioventricular septal defect (AVSD). Gene ontology (GO) enrichment analysis was used to examine the biological processes of the protein modules associated with CHD phenotypes. We observed six modules that were significantly correlated with the clinical phenotype clusters. Cluster 1 was significantly associated with protein modules 4 and 8, and was enriched in carbohydrate metabolism, glutathione metabolism, and immune response. Cluster 2 was significantly associated with protein modules 9 and 10 and was enriched for ECM receptor response, protein transport, and signal transduction. Cluster 3 was significantly associated with protein modules 2 and 3 and was mostly enriched for the regulation of myocardial growth, development, and regulation of the actin cytoskeleton (Fig 4B). To further investigate potential factors in different clusters affecting disease occurrence, we analyzed the PPI network for pathways (Fig 4B; Table EV4). The data revealed key molecules in each pathway, such as amylase alpha 2A (AMY2A), immunoglobulin lambda‐like polypeptide 5 (IGLL5), MYL9, integrin subunit alpha 1 (ITGA1), RAB6A, member RAS oncogene family (RAB6A), and catenin beta 1 (CTNNB1) (Fig 4C). These hub genes may differentiate CHD from control groups.

Furthermore, we investigated the relationship between 10 protein modules and 18 clinical indices, with only three modules (M2, M3, and M6) showing strong correlations with CHD pathology. Cluster 3 (M2 and M3) was positively associated with blood lipids and positively linked to thyroid hormones. These results further indicate that an increase in blood lipids and thyroid hormone levels may be a risk factor for CHD. In addition, M6 levels were negatively correlated with thyroxine hormone levels (*P* < 0.05) (Fig 4D). Furthermore, we compared the concentration of seven kinds of lipids in the plasma of women in early gestation among the control and different clusters of CHDs and found that the level of free fatty acids was significantly downregulated in the CHD group, while the other six lipids, including low‐density lipoprotein (LDL), triglyceride, high‐density lipoprotein, total cholesterol (TC), apolipoprotein A (APOA), and apolipoprotein B (APOB), were upregulated in the CHD group (Fig 4E). These results were consistent with previous findings that lipids are major risk factors for CHD (Pencina *et al*, 2019; Bogachkov *et al*, 2020; Hu *et al*, 2020; Kalaivani & Jaleel, 2020; Mehta *et al*, 2020; Dugani *et al*, 2021), indicating that fluctuations in blood lipids may be associated with the occurrence of CHD. Since we were specifically interested in protein module 2 from the WGCNA analysis, we further examined the DEPs between CHD and control samples. Of these 26 proteins in module 2, two proteins were positively associated with the level of blood lipids (Fig 4F), and SELENBP1 and MSN were significantly increased in cluster 3 of CHD, such that SELENBP1 increased by 51% (*P* = 0.0114) and MSN increased by 201% (*P* = 0.0162).

### Immune landscape of CHD

To investigate changes in the immune microenvironment after CHD occurrence, we employed xCell to generate cell‐type immune enrichment scores based on the proteome. Here, we identified 21 different cell types related to immune (CD4^+^ T cells and NK cells) or stromal (adipocytes and endothelial cells) signatures, and discriminated each sample by the presence or absence of specific cell types (Fig 4G). Endothelial cells, myocytes, and pericytes were enriched in the control group, whereas CD4^+^ T cells, adipocytes, and preadipocytes were enriched in the CHD group (Fig 4G). Specifically, CD4^+^ T cells and preadipocytes were enriched in cluster 3, while adipocytes and preadipocytes were enriched in cluster 2 (Fig 4G and H). These results indicate that CHD occurrence is accompanied by an increase in blood lipids, adipocytes, and CD4^+^ T cells and a reduction in endothelial cells, myocytes, and pericytes, which may be involved in the pathogenesis of CHD, as shown in Fig 4I. An increase in blood lipids induces CHD by inhibiting the proliferation and migration of cardiomyocytes, hindering angiogenesis, and activating CD4^+^ T cells. Activated CD4^+^ T cells can release proinflammatory cytokines to activate macrophages and vascular cells and cause acute inflammation. They can also cause a chronic inflammatory state, which may further damage heart development (Fig 4I).

### Identification of biomarker combinations for the recognition of CHD based on machine learning

**Figure 5:**
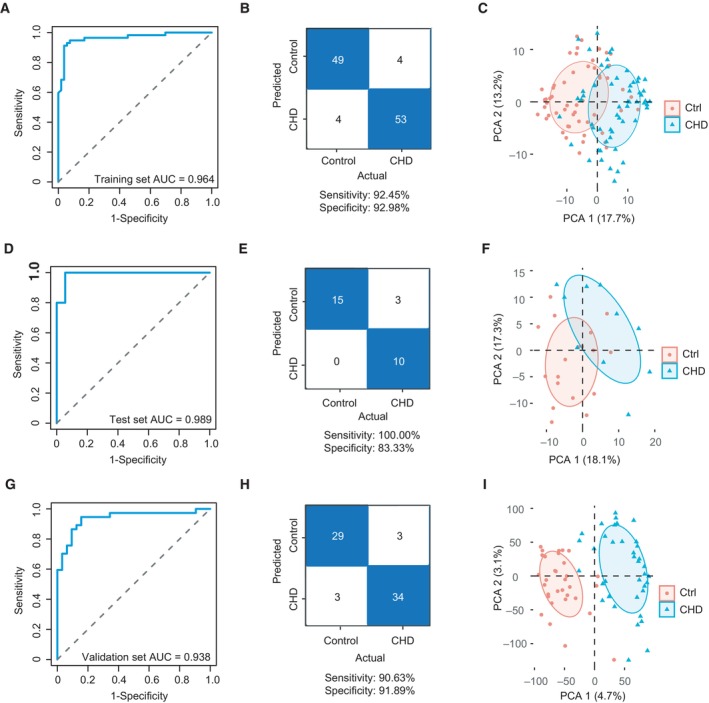
Exploiting machine learning for the development of biomarker combinations to predict CHD A–C(A) The receiver operating characteristic (ROC) curve for the training dataset of group 1, (B) Confusion matrix for the combination biomarkers in the training dataset, and (C) principal component analysis (PCA) plot for the prediction of CHD and control outcomes.D–F(D) The ROC curve for the test dataset of group 1, (E) confusion matrix for the combination biomarkers in the test dataset, and (F) PCA plot for the prediction of CHD and control outcomes.G–I(G) The ROC curve for the external validation set in group 2, (H) confusion matrix for the combination biomarkers in the external validation set, and (I) PCA plot for the prediction of CHD and control outcomes.

**Figure EV3:**
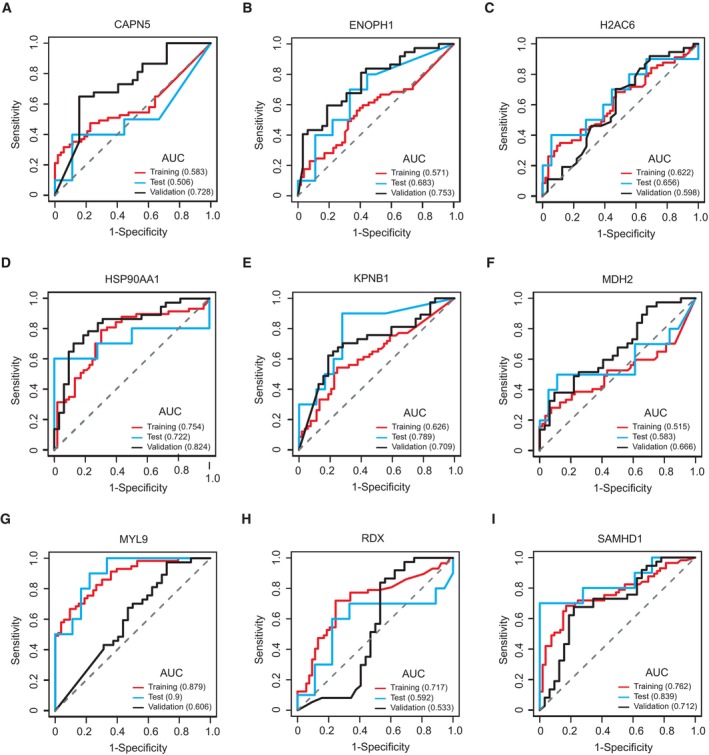
CHD diagnostic performance of nine candidate biomarkers The receiver operating characteristic (ROC) curve of protein calpain‐5 (CAPN5) in the training set, test set, and validation set.The ROC curve of protein enolase‐phosphatase E1 (ENOPH1) in the training set, test set, and validation set.The ROC curve of protein histone H2A type 1‐C (H2AC6) in the training set, test set, and validation set.The ROC curve of protein heat shock protein HSP 90‐alpha (HSP90AA1) in the training set, test set, and validation set.The ROC curve of protein importin subunit beta‐1 (KPNB1) in the training set, test set, and validation set.The ROC curve of protein malate dehydrogenase (MDH2) in the training set, test set, and validation set.The ROC curve of protein myosin regulatory light polypeptide 9 (MYL9) in the training set, test set, and validation set.The ROC curve of protein radixin (RDX) in the training set, test set, and validation set.The ROC curve of protein deoxynucleoside triphosphate triphosphohydrolase 1 (SAMHD1) in the training set, test set, and validation set.

Based on the plasma proteomics data in group 1, we used a machine learning approach to identify potential biomarker combinations to recognize fetuses with CHD in pregnant women during the first trimester. The selection criteria for potential biomarkers are described in the Materials and Methods. To optimize the parameters for the training model and assess model performance, we conducted 10‐fold cross‐validation. The machine learning model was established based on 80% proteomics data from group 1, selecting a biomarker combination containing the following nine proteins: calpain‐5 (CAPN5), enolase‐phosphatase E1 (ENOPH1), histone H2A type 1‐C (H2AC6), HSP90AA1, importin subunit beta‐1 (KPNB1), MDH2, MYL9, radixin (RDX), and SAMHD1. This model achieved an area under the curve (AUC) of 0.964 (95% confidence interval [CI] = 0.862–0.968) in the training set (Fig 5A). The remaining data in group 1 were used for the test dataset, which reached an AUC value of 0.989 (95% CI = 0.718–0.977) (Fig 5D). These results suggested the clinical significance of this combination of biomarkers for the diagnosis of CHD based on maternal plasma samples. To further verify the performance of this combination, we collected 69 plasma samples from group 2 for inter‐group validation (32 control and 37 CHD cases), with an AUC value of 0.963 (95% CI = 0.878–0.997) (Fig 5G). Furthermore, evaluating the reliability of the machine‐learning strategy, the results of confusion matrices and PCA of these biomarker combinations showed relatively high accuracy in classification of the control and CHD groups (Fig 5B, C, E, F, H and I). The potential clinical significance for the recognition of CHD using individual biomarkers is shown in Fig EV3.

## Discussion

Owing to the recent improvement in strategies for cardiac interventional therapy and surgery, CHD, which can be life‐threatening for neonates, seems less lethal. The key to better management of CHD, particularly the critical cases, relies on early diagnosis and prompt treatment, which can even be conducted prenatally through fetal cardiac intervention for aortic stenosis, thus avoiding the development of hypoplastic left heart syndrome. Therefore, prenatal diagnosis of CHD not only prepares the parents and medical staff for treatment after birth but also allows early intervention and may yield a better prognosis. The well‐established prenatal diagnosis of CHD is the second‐trimester anomaly scan (SAS) during pregnancy based on fetal echocardiography. The detection rate of CHD using SAS in unselected populations is approximately 45% and varies among different types of CHD, for example, 90.8% for tricuspid valve atresia and 22.3% for coarctation of the aorta (van Velzen *et al*, 2018), and affected by personal experience. As fetal heart development is completed around the 6^th^ week of pregnancy (Helle & Priest, 2020), objective indices that can recognize CHD before the heart is fully developed are needed for accurate prenatal diagnosis and future prenatal interventions.

Several hematological biomarkers, including PAPP‐A, free β‐HCG, natriuretic peptide, and chromosome microarray analysis, have potential value in the early detection of CHD (Miyoshi *et al*, 2018; Wang *et al*, 2018b; Alanen *et al*, 2019; Gu *et al*, 2019; Bahado‐Singh *et al*, 2020; Chen *et al*, 2022). In this study, performed in two groups from two different hospitals, we collected plasma samples from first‐trimester pregnant women carrying fetuses with or without CHD. This study design aimed to identify maternal protein expression profiles which could be used as novel non‐invasive biomarkers for the early detection of fetal CHD. Here, we identified 184 significantly upregulated proteins and 213 downregulated proteins in the CHD group in group 1 and 80 upregulated proteins and 145 downregulated proteins in group 2, and the Venn diagram revealed 25 upregulated proteins and 49 downregulated proteins in both the groups, indicating that the maternal plasma protein profile at early pregnancy has the potential to reflect the onset of CHD. We identified 10 protein modules using WGCNA and classified CHD subtypes into three clusters using hierarchical clustering analysis and found six modules that significantly correlated with the clinical phenotype clusters, suggesting a potential common pathogenesis mechanism in each cluster. Moreover, to identify potential biomarkers for the accurate identification of fetal CHD, we developed a machine‐learning‐based pipeline, resulting in the identification of seven biomarkers as well as a set of biomarker combinations that could accurately predict different CHD outcomes, with a high degree of sensitivity.

In addition, our study revealed DEPs involved in metabolism among patients with CHDs and controls. This is consistent with the fact that the dysregulation of metabolism, such as maternal diabetes (Lisowski *et al*, 2010), is associated with the risk of CHD. Recently, maternal obesity has also been identified as a risk factor for CHD (Persson *et al*, 2019). Since the phenotype overlaps between obesity and diabetes, it is yet to be elucidated whether obesity increases the CHD risk mediated by promoting diabetes, or whether it damages the developing heart through dyslipidemia and other secondary effects including hyperinsulinemia, insulin resistance, and oxidative stress (Helle & Priest, 2020; Chen *et al*, 2023a). Here, we found lipids, including LDL and total TC, were upregulated in the CHD group, and the cell‐type immune enrichment suggested the enrichment of adipocytes and pre‐adipocytes in the CHD group. This indicates that dyslipidemia might contribute to CHD. Immune CD4^+^ T cells were also enriched in the CHD group. Existing knowledge of CHD coinciding with immune and inflammatory associations is limited. Several immune profiles are associated with CHDs (Singampalli *et al*, 2021), and the altered immune/inflammation state might be the cause of CHD (Swirski & Nahrendorf, 2018), the result of CHDs, or accompanied by CHDs sharing a common etiology. As CD4^+^ T cells can be modulated by adipocyte‐derived lipids, they may play an active role in the occurrence of CHD among obese individuals.

Among the limitations of this study, first, we cannot exclude the potential impact of preclinical conditions on the protein profile result. Pregnant women recruited in this study were confirmed not to have obvious health problems or severe metabolic disorders. However, there might be certain preclinical conditions undetected which can be exacerbated by pregnancy and thus disturb the protein profile results. Another set of proteomics data from the same cohort before or after pregnancy would be needed in future studies to eliminate this possibility. Second, this study should be applied with caution to predict critical CHDs. In our centers, most critical CHD cases were detected during early or mid‐gestation by echocardiogram and aborted according to parents' will, leaving no chance for genetic study or postnatal phenotype confirmation. As we only recruited cases who had a complete physical examination, echocardiogram, and genetic study by neonatologists and pediatric cardiologists after birth, the cases studied were mainly mild VSD and ASD, leaving insufficient data to conduct stratified analysis to assess the specificity and sensitivity according to the severity of the disease, which is certainly important to the clinical settings. Further studies with enlarged sample sizes or recruiting critical CHDs specifically would help to address this problem. Also, due to the limited sample size and the low prevalence of CHD in the general population, the conclusion should be drawn with caution and the future administration of this omic approach should be conducted carefully. Trials in larger scales are needed to verify the conclusion and test the diagnostic panel before it can be applied in clinical settings. Besides, in our study, cases present higher lipid values compared with controls, thus the correlation of proteins involved in lipid metabolism to the case group might reflect overfitting. Further studies comparing the lipid composition and proteins involved in lipid metabolism between case and control groups on larger scales and revealing the underlying mechanism would help to understand the role of lipid metabolism in the occurrence of CHD.

In conclusion, we identified and validated a biomarker combination containing nine proteins that can serve as novel non‐invasive biomarkers for the maternal detection of fetal CHD. Only 2 μl plasma from pregnant women at early gestation would allow the administration of this omic approach and guide mom to be with a high risk of having CHD offspring to receive more intensive follow‐up and prepare for prenatal intervention, which certainly helps to the targeted use of medical resources. Also, this work provides a highly valuable proteomics resource for the research community to better understand the cause of CHD, identify a series of valuable biomarker candidates, and provide clues for potential therapeutic strategies.

## Materials and Methods

### Study participants and ethics

In this study, maternal plasma samples during early pregnancy (10–12 weeks gestation) from two independent case–control groups were analyzed. The design and conduct of the study was approved and supervised by the Ethics Committee of the Obstetrics and Gynecology Hospital of Fudan University through Ethic Vote 2015‐17‐C1 and the Ethics Committee of the International Peace Maternity & Child Health Hospital of China Welfare Institute through Ethic Vote (GKLW)‐2018‐37, in accordance with the criteria established by the Declaration of Helsinki, and all experiments conformed to the Department of Health and Human Services Belmont Report. Written informed consent was obtained from all human subjects.

Group 1 was recruited from the Obstetrics & Gynecology Hospital of Fudan University in Shanghai, China, from January 2018 to December 2019, as described previously (Zhang *et al*, 2022). This group included 67 pregnant women whose babies were later diagnosed with CHD and 71 controls with healthy offspring. Group 2 consisting of 37 cases and 32 controls from the International Peace Maternity and Child Health Hospital of the China Welfare Institute were recruited and analyzed independently and concurrently. Pregnant women included were in good health when recruited in our study, and the pregnant women in the control group were chosen on the basis of matching the general health status, which was also confirmed by the baseline comparison of their vital signs and physiological indexes. Pregnant women with one of the following situations were excluded: clinical or biochemical signs of infection, multiple gestations, diabetes mellitus, or other severe metabolic disorders. CHD phenotypes were first identified by examining malformations during week 22 of gestation and were confirmed after birth using color echocardiography. Cases of isolated patent ductus arteriosus, patent foramen ovale, bicuspid aortic valve, coronary anomalies, as well as CHD that relate mainly to the vascular system were excluded from the present study. All patients with genetic syndromes or known chromosomal abnormalities (e.g., Down's syndrome, Holt–Oram syndrome, Alagille syndrome, DiGeorge syndrome, William syndrome, and Noonan syndrome) or family history of CHD in a first‐degree relative (parent, sibling, or child) were excluded. Also, cases combined with other non‐cardiovascular malformations, tumors, or systematic diseases were not recruited. All participants were unrelated ethnic Han Chinese. The investigators conducting the proteomic study were blind to the recruitment. The demographic characteristics of pregnant women bearing children either with or without CHD, including the use of folic acid, maternal/paternal smoking and drinking status, blood biochemical index, and the phenotypes of the CHD offspring, are shown in Table 1 and Dataset EV1.

**Table 1:** Demographic characteristics of participants.

	Group 1			Group 2		
	Control (*n* = 71)	Case (*n* = 67)	*P*‐value	Control (*n* = 32)	Case (*n* = 37)	*P*‐value
Pregnant characteristics						
Age, years	30.69 ± 3.52	30.97 ± 3.58	0.64	31.32 ± 3.64	31.00 ± 3.53	0.71
Gestational week	10.99 ± 0.73	10.99 ± 0.77	0.99	10.97 ± 0.74	10.95 ± 0.70	0.90
BMI	21.32 ± 2.08	21.42 ± 2.32	0.80	21.22 ± 2.32	21.25 ± 2.43	0.96
Fasting glucose, mmol/l	4.52 ± 0.28	4.52 ± 0.39	0.94	4.57 ± 0.42	4.44 ± 0.29	0.13
Serum cholesterol, mmol/l	4.41 ± 0.78	5.66 ± 1.54	1.18E‐08	4.33 ± 0.76	5.23 ± 1.51	3.06E‐03
Serum triglyceride, mmol/l	1.31 ± 0.49	2.41 ± 1.46	1.42E‐08	2.04 ± 1.54	1.14 ± 0.35	1.91E‐03
Serum folate, ng/ml	16.87 ± 2.50	16.46 ± 3.10	0.40	17.19 ± 1.85	16.76 ± 1.77	0.33
Serum vitamin B_12_, pg/ml	481.97 ± 142.26	532.79 ± 259.46	0.16	506.94 ± 173.11	584.92 ± 186.44	0.08
Serum vitamin D, ng/ml	17.67 ± 7.53	17.74 ± 8.34	0.96	19.56 ± 7.47	16.25 ± 6.02	0.06
Serum homocysteine, μmol/l	6.54 ± 1.44	6.34 ± 1.14	0.38	6.37 ± 0.98	6.66 ± 1.33	0.30
Smoking status			0.953			0.881
Yes	2	2		2	2	
No	69	65		30	35	
Drinking status			NA			NA
Yes	0	0		0	0	
No	71	67		32	37	
Use of folic acid			0.875			0.916
Yes	50	48		22	25	
No	21	19		10	12	
Offspring phenotypes						
VSD		33			16	
ASD		2			18	
PLSVC		8				
PS		6				
LVOTO		6				
AVSD		2				
AS		4				
TR					3	
PTA		2				
TOF		2				
TGA		1				
RVOTO		1				

Data presented are given in mean ± SD. *P*‐values in pregnant characteristics were derived from unpaired two‐sample *t*‐test (two groups have the same SD) or unpaired two‐sample *t*‐test with Welch's correction (two groups do not have the equal SD); *P*‐values in offspring characteristics were derived from Chi‐square test. VSD, ventricular septal defect; ASD, atrial septal defect; PLSVC, persistent left superior vena cava; PS, pulmonary stenosis; LVOTO, left ventricular outflow track obstruction; AVSD, atrioventricular septal defect; AS, aortic stenosis; TR, tricuspid regurgitation; PTA, persistent truncus arteriosus; TOF, Tetralogy of Fallot; TGA, transposition of the great arteries; RVOTO, right ventricular outflow tract obstruction; NA, not available.

### Sample preparation

For protein extraction from plasma samples, the top 14 highest abundance plasma proteins were firstly removed using an immunodepleting kit (Thermo Fisher) according to the manufacturer's instructions, and then inactivated at 85°C for 10 min. The depleted plasma was digested by trypsin at an enzyme‐to‐protein mass ratio of 1:25 overnight at 37°C, and the peptides were then extracted and dried (SpeedVac, Eppendorf).

### Liquid chromatography–tandem mass spectrometry analysis

Liquid chromatography–tandem mass spectrometry (LC–MS/MS) analysis was performed on an EASY‐nLC 1200 ultra‐high‐pressure system coupled to an Orbitrap Fusion Lumos mass spectrometer via a nano‐electrospray ion source (Thermo Fisher Scientific).

The dried peptides were dissolved in 12 μl loading buffer (0.1% formic acid in water), and 5 μl of this sample was loaded onto a trap column (C18, 100 μm I.D., 2.5 cm) at a maximum pressure of 280 bar with 14 μl of solution A (0.1% formic acid in water). The peptides were separated on a 150 μm I.D. × 15 cm column (C18, 1.9 μm, 120 Å, Maisch GmbH) with a linear gradient of 15–30% mobile phase B (acetonitrile and 0.1% formic acid) at a flow rate of 600 nl/min for 75 min.

The MS data were obtained in the DIA scan mode. The DIA method consisted of an MS1 scan from 300 to 1,400 *m/z* at 60 K resolution (Automatic Gain Control [AGC] target 4e5 or 50 ms). Thirty DIA segments were acquired sequentially at 15 K resolution, with an AGC target of 5e4 or 22 ms for the maximal injection time. The setting “inject ions for all available parallelizable times” was enabled. The higher‐energy collision‐induced dissociation fragmentation was set to a normalized collision energy of 30%, and the default charge state for MS2 was set to 3.

### Mass spectrometry data processing

All data were processed using Firmiana (Feng *et al*, 2017), and DIA data were searched against the UniProt human protein database (updated on 2019.12.17, 20,406 entries) using FragPipe (v12.1) and MSFragger (2.2) (Kong *et al*, 2017). The trypsin proteolytic cleavage rule was used, permitting a maximum of two missed cleavages and a minimum peptide length of seven amino acids. The search included cysteine carbamidomethylation as a fixed modification and N‐acetylation and oxidation of methionine as variable modifications. Peptides were identified with a precursor mass accuracy deviation of 20 ppm and fragment mass deviation of 50 mmu. The precursor ion score charges were set to +2, +3, and +4. The false discovery rate (FDR) was 1% for both the protein and peptide levels. A total of 327 libraries were used as reference spectra libraries, and the DIA results were merged into these reference libraries using the SpectraST software.

Data‐independent acquisition data were analyzed using DIA‐NN (v1.7.0) (Demichev *et al*, 2020). The default settings were used for DIA‐NN (precursor FDR: 5%, Log lev: 1, mass accuracy: 20 ppm, MS1 accuracy: 10 ppm, scan window: 30, implicit protein group: genes, and quantification strategy: robust LC (high accuracy)). The identified peptides were quantified as the average of the chromatographic fragment ion peak areas across all reference libraries. Protein quantification was performed using the label‐free and intensity‐based absolute quantification (iBAQ) approach (Zhang *et al*, 2012). The peak area was calculated as a part of the corresponding proteins. The fraction of total (FOT) was used to represent the normalized abundance of a particular protein across the samples. FOT was defined as the iBAQ of a protein divided by the total iBAQ of all identified proteins within a sample. The FOT values were multiplied by 10^5^ for ease of presentation, and missing values were imputed at one‐tenth of the minimum value.

### Bioinformatics analysis

#### Missing value imputation

For the plasma proteomic data, FOTs multiplied by 10^5^ were used for quantification, and missing values were imputed with one‐tenth of the minimum value.

#### Statistical analysis

Data statistical analysis was performed with GraphPad Prism 8 software and R‐studio scripts (version 1.3.1093). After missing value imputations and data normalization, significance analysis was performed using Student's *t*‐test to identify differentially expressed proteins between CHD and healthy controls. Correlations were tested using Pearson correlation coefficients. Calculation of Pearson correlation scores and associated *P*‐values of protein intensities to thyroid hormone, blood lipids, and blood glucose was performed in R.

#### Differentially expressed proteins

Bioinformatics analysis was performed using R‐studio scripts (version 1.3.1093). Only proteins with < 50% NAs (missing values) were considered for differential expression analysis. The relative abundances were log_2_ transformed for each protein to obtain the final relative abundance values, and Student's *t*‐test was used to determine the significantly changed proteins between the cases and controls in groups 1 and 2. Significant DEPs with *P* < 0.05 as well as a fold change > 2 or < 1/2 were labeled as upregulated and downregulated genes, respectively. Differential expression is presented in volcano plots.

#### Functional enrichment analysis

DAVID Bioinformatics Resources 6.8 (http://david.ncifcrf.gov, an online bioinformatics tool for gene function annotation) and Reactome (https://reactome.org) were used to analyze the functional enrichment of the DEPs between the case and control samples (Huang da *et al*, 2009). The pathways with *P* < 0.05 were considered statistically significant. PPIs were visualized using String 11.0 and Cytoscape.

#### Weighted gene co‐expression network analysis

The R package WGCNA (Langfelder & Horvath, 2008) was used to construct protein co‐expression networks. We input 2,280 proteins present in more than 30% of the 208 patients into WGCNA. The Spearman correlation coefficient between protein expression profiles using the block‐wise module WGCNA function with the following settings: soft threshold power beta = 5 (as it was the smallest threshold resulting in a scale‐free R2 fit of 0.85), minimum module size = 20, and merge cut height = 0.3, which calculated topologic overlap (TOM) with bicor correlation function. Furthermore, genes were hierarchically clustered using 1‐TOM (dissTOM) as the distance measure. Each module was summarized by the first principal component of the scaled module expression profiles, termed module eigengene.

#### Cell‐type enrichment analyses

The abundance of each cell type was inferred using the x‐Cell (http://xcell.ucsf.edu) tool (Aran *et al*, 2017), which performed cell‐type enrichment analysis from gene expression data for 64 immune and stromal cell types, generating an immune score per sample.

#### Potential diagnostic biomarkers for CHD

Data processing and machine learning were performed using R‐Studio scripts (version 1.3.1093). We defined 80% of group 1 as the training set and 20% of the remaining data as the test set. Features were selected using a random forest (*n*_trees = 1,000). To avoid overfitting, the number of protein types in the combination should be considerably lesser than the number of samples. Therefore, the combinations of biomarkers with fewer than 10 proteins were selected and optimized. In addition, plasma samples from group 2 were set as external validation sets. The test set and external validation set were used to evaluate candidate diagnostic biomarkers. To assess the sensitivity and specificity of the model, 10‐fold cross‐validation and 10 repeats were applied.

#### Western blotting

The western blotting assays were performed as described before (Wang *et al*, 2018a; Zhang *et al*, 2021b). Briefly, 80 μl of plasma was mixed with 20 μl SDS loading buffer followed by the standard immunoblotting procedures. The primary antibodies used for western blot analysis are listed as follows: anti‐SHMT1 (dilution 1:1,000, #80715S, CST), anti‐G6PD (dilution 1:1,000, 25413‐1‐AP, Proteintech), and anti‐MYL9 (dilution 1:1,000, A8738, ABclonal). Protein abundance was detected by measuring chemiluminescence on Typhoon FLA 9500 (GE Healthcare, Little Chalfont, UK).

## Author contributions

**Ya‐Nan Yin:** Data curation; formal analysis; funding acquisition; investigation; visualization; writing – original draft; writing – review and editing. **Li Cao:** Conceptualization; resources; data curation; validation; writing – original draft; project administration; writing – review and editing. **Jie Wang:** Validation; investigation; visualization; writing – original draft; project administration; writing – review and editing. **Yu‐Ling Chen:** Data curation; investigation; writing – original draft; writing – review and editing. **Hai‐Ou Yang:** Conceptualization; resources; software; methodology; writing – original draft. **Su‐Bei Tan:** Formal analysis; investigation. **Ke Cai:** Data curation; formal analysis; investigation. **Zhe‐Qi Chen:** Software; investigation. **Jie Xiang:** Data curation; formal analysis. **Yuan‐Xin Yang:** Investigation. **Hao‐Ran Geng:** Investigation. **Ze‐Yu Zhou:** Investigation. **An‐Na Shen:** Investigation. **Xiang‐Yu Zhou:** Investigation. **Yan Shi:** Investigation; methodology. **Rui Zhao:** Conceptualization; formal analysis; supervision; project administration; writing – review and editing. **Kun Sun:** Conceptualization; supervision; project administration; writing – review and editing. **Chen Ding:** Conceptualization; supervision; project administration; writing – review and editing. **Jian‐Yuan Zhao:** Conceptualization; data curation; supervision; project administration; writing – review and editing.

## Disclosure and competing interests statement

The authors declare that they have no conflict of interest.

## For more information

- Jian‐Yuan Zhao's Website: https://www.x‐mol.com/groups/jianyuan_zhao.
- Chen Ding's Website: https://hupi.fudan.edu.cn/rcdw/rc_content.jsp?urltype=news.NewsContentUrl&wbtreeid=1122&wbnewsid=2099.
- Proteome resources: https://www.iprox.cn/page/home.html.

## Supporting information



Expanded View Figures PDF

Table EV1

Table EV2

Table EV3

Table EV4

Dataset EV1

Dataset EV2

Dataset EV3

Dataset EV4

Dataset EV5

Dataset EV6

PDF+

Source Data for Figure 1

Source Data for Figure 2

Source Data for Figure 3

Source Data for Figure 4

## Data Availability

The datasets produced in this study are available in the following databases: Proteomics data: iProX IPX0005331001 (https://www.iprox.cn/page/project.html?id=IPX0005331001).
