# Supplementary material for: Proteome profiling of early gestational plasma reveals novel biomarkers of congenital heart disease
Source: EMBO Mol Med. 2023 Oct 16;15(12):e17745. doi: 10.15252/emmm.202317745 (PMC10701625; doi:10.15252/emmm.202317745)
Supplement: Supplementary file 1 — Expanded View Figures PDF [file EMMM-15-e17745-s008.pdf]

Expanded View Figures

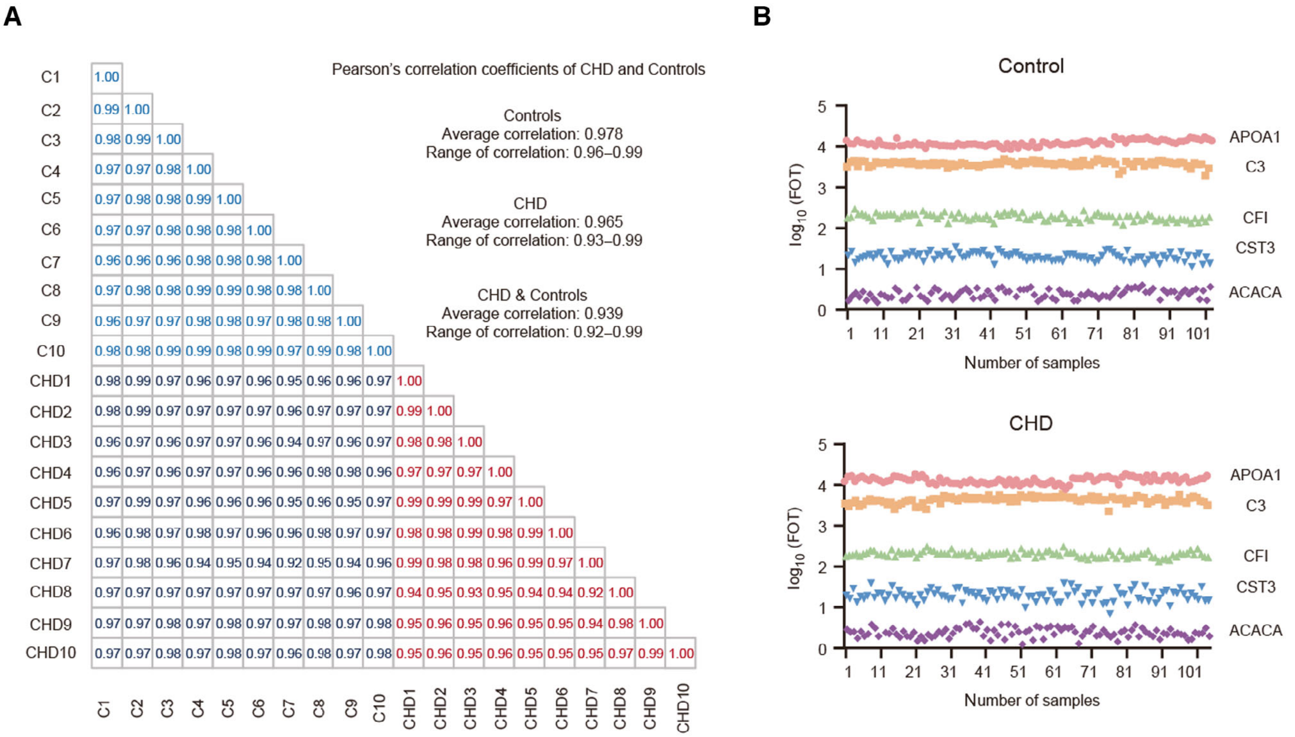

Figure EV1. Reproducibility of plasma data.

A Pearson's correlation coefficients for replicate proteome profiling of 20 plasma samples (10 CHD and 10 healthy control samples).

B Reproducibility of the fraction of total (FOT) of six proteins in 207 samples. FOT was defined as the iBAQ of a protein divided by the total iBAQ of all identified proteins within a sample.

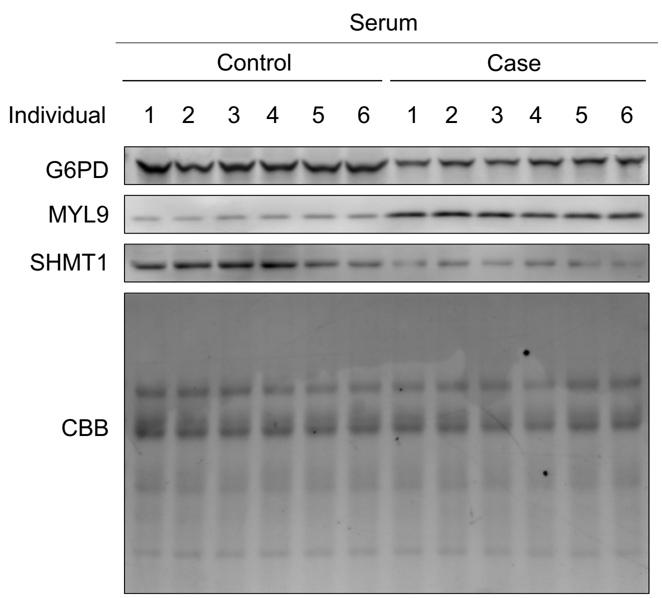

Figure EV2. Protein levels in serum from pregnant women with CHD or normal offspring.

Figure EV3. CHD diagnostic performance of nine candidate biomarkers.

A The receiver operating characteristic (ROC) curve of protein calpain-5 (CAPN5) in the training set, test set, and validation set.
B The ROC curve of protein enolase-phosphatase E1 (ENOPH1) in the training set, test set, and validation set.
C The ROC curve of protein histone H2A type 1-C (H2AC6) in the training set, test set, and validation set.
D The ROC curve of protein heat shock protein HSP 90-alpha (HSP90AA1) in the training set, test set, and validation set.
E The ROC curve of protein importin subunit beta-1 (KPNB1) in the training set, test set, and validation set.
F The ROC curve of protein malate dehydrogenase (MDH2) in the training set, test set, and validation set.
G The ROC curve of protein myosin regulatory light polypeptide 9 (MYL9) in the training set, test set, and validation set.
H The ROC curve of protein radixin (RDX) in the training set, test set, and validation set.
I The ROC curve of protein deoxynucleoside triphosphate triphosphohydrolase 1 (SAMHD1) in the training set, test set, and validation set.

© 2023 The Authors

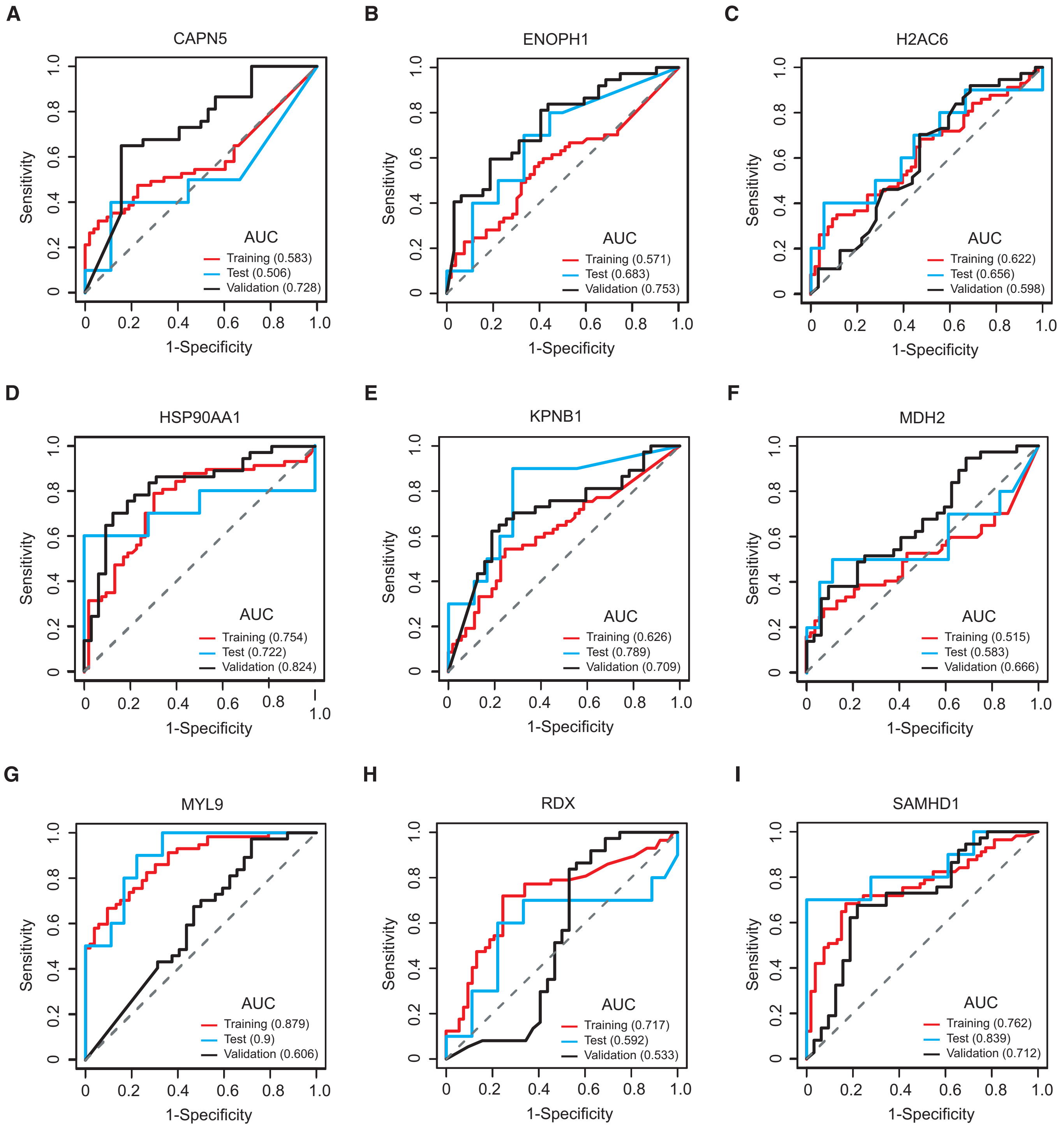
A
CAPN5
Sensitivity
1-Specificity
AUC
Training (0.583)
Test (0.506)
Validation (0.728)
B
ENOPH1
Training (0.571)
Test (0.683)
Validation (0.753)
C
H2AC6
Training (0.622)
Test (0.656)
Validation (0.598)
D
HSP90AA1
Training (0.754)
Test (0.722)
Validation (0.824)
E
KPNB1
Training (0.626)
Test (0.789)
Validation (0.709)
F
MDH2
Training (0.515)
Test (0.583)
Validation (0.666)
G
MYL9
Training (0.879)
Test (0.9)
Validation (0.606)
H
RDX
Training (0.717)
Test (0.592)
Validation (0.533)
I
SAMHD1
Training (0.762)
Test (0.839)
Validation (0.712)

Figure EV3.
