# Supplementary material for: Proteome profiling of early gestational plasma reveals novel biomarkers of congenital heart disease
Source: EMBO Mol Med. 2023 Oct 16;15(12):e17745. doi: 10.15252/emmm.202317745 (PMC10701625; doi:10.15252/emmm.202317745)
Supplement: Supplementary file 17 [file EMMM-15-e17745-s003.pdf]

EMBO Molecular Medicine

Proteome profiling of early gestational plasma reveals novel biomarkers of congenital heart disease

Yanan Yin, Li Cao, Jie Wang, Yu-Ling Chen, Hai-Ou Yang, Su-Bei Tan, Ke Cai, Zhe-Qi Chen, Jie Xiang, Yuan-Xin Yang, Hao-Ran Geng, Ze-Yu Zhou, An-Na Shen, Xiang-Yu Zhou, Yan Shi, Rui Zhao, Kun Sun, Chen Ding, and Jian-Yuan Zhao
DOI: 10.15252/emmm.202317745

Corresponding authors: Jian-Yuan Zhao (zhaojy@fudan.edu.cn) , Kun Sun (sunkun@xinhuamed.com.cn), Rui Zhao (14111510019@fudan.edu.cn), Chen Ding (chend@fudan.edu.cn)

Review Timeline:

Submission Date:	6th Apr 23
Editorial Decision:	26th May 23
Revision Received:	24th Aug 23
Editorial Decision:	13th Sep 23
Revision Received:	26th Sep 23
Accepted:	27th Sep 23

Editor: Zeljko Durdevic

Transaction Report:

(Note: With the exception of the correction of typographical or spelling errors that could be a source of ambiguity, letters and reports are not edited. Depending on transfer agreements, referee reports obtained elsewhere may or may not be included in this compilation. Referee reports are anonymous unless the Referee chooses to sign their reports.)

26th May 2023

Dear Prof. Zhao,

Thank you for the submission of your manuscript to EMBO Molecular Medicine. We have now received feedback from the three reviewers who agreed to evaluate your manuscript. As you will see from the reports below, all referees recognize potential interest of the study, but also raise serious concerns that should be addressed in a major revision. The referee #1 point about "another set of proteomics data from same cohort before or after pregnancy will be very helpful to rule out this possibility" should be addressed in discussion as a limitation of the study. If you would like to discuss further the points raised by the referees, I am available to do so via email or video. Let me know if you are interested in this option.

Further consideration of a revision that addresses reviewers' concerns in full will entail a second round of review. EMBO Molecular Medicine encourages a single round of revision only and therefore, acceptance or rejection of the manuscript will depend on the completeness of your responses included in the next, final version of the manuscript. For this reason, and to save you from any frustrations in the end, I would strongly advise against returning an incomplete revision.

We would welcome the submission of a revised version within three months for further consideration. Please let us know if you require longer to complete the revision.

I look forward to receiving your revised manuscript.

Yours sincerely,

Zeljko Durdevic

Zeljko Durdevic
Editor
EMBO Molecular Medicine

*****

When submitting your revised manuscript, please carefully review the instructions that follow below. We perform an initial quality control of all revised manuscripts before re-review; failure to include requested items will delay the evaluation of your revision.

We require:

1) A .docx formatted version of the manuscript text (including legends for main figures, EV figures and tables). Please make sure that the changes are highlighted to be clearly visible.

2) Individual production quality figure files as .eps, .tif, .jpg (one file per figure). For guidance, download the 'Figure Guide PDF': (https://www.embopress.org/page/journal/17574684/authorguide#figureformat).

3) A .docx formatted letter INCLUDING the reviewers' reports and your detailed point-by-point responses to their comments. As part of the EMBO Press transparent editorial process, the point-by-point response is part of the Review Process File (RPF), which will be published alongside your paper.

4) A complete author checklist, which you can download from our author guidelines (https://www.embopress.org/page/journal/17574684/authorguide#submissionofrevisions). Please insert information in the checklist that is also reflected in the manuscript. The completed author checklist will also be part of the RPF.

5) Please note that all corresponding authors are required to supply an ORCID ID for their name upon submission of a revised manuscript.

6) It is mandatory to include a 'Data Availability' section after the Materials and Methods. Before submitting your revision, primary datasets produced in this study need to be deposited in an appropriate public database, and the accession numbers and database listed under 'Data Availability'. Please remember to provide a reviewer password if the datasets are not yet public (see https://www.embopress.org/page/journal/17574684/authorguide#dataavailability).

In case you have no data that requires deposition in a public database, please state so in this section. Note that the Data Availability Section is restricted to new primary data that are part of this study.

7) For data quantification: please specify the name of the statistical test used to generate error bars and P values, the number (n) of independent experiments (specify technical or biological replicates) underlying each data point and the test used to calculate p-values in each figure legend. The figure legends should contain a basic description of n, P and the test applied. Graphs must include a description of the bars and the error bars (s.d., s.e.m.). See also 'Figure Legend' guidelines: https://www.embopress.org/page/journal/17574684/authorguide#figureformat

8) At EMBO Press we ask authors to provide source data for the main manuscript figures. Our source data coordinator will contact you to discuss which figure panels we would need source data for and will also provide you with helpful tips on how to upload and organize the files.

9) Our journal encourages inclusion of *data citations in the reference list* to directly cite datasets that were re-used and obtained from public databases. Data citations in the article text are distinct from normal bibliographical citations and should directly link to the database records from which the data can be accessed. In the main text, data citations are formatted as follows: "Data ref: Smith et al, 2001" or "Data ref: NCBI Sequence Read Archive PRJNA342805, 2017". In the Reference list, data citations must be labeled with "[DATASET]". A data reference must provide the database name, accession number/identifiers and a resolvable link to the landing page from which the data can be accessed at the end of the reference. Further instructions are available at .

10) We replaced Supplementary Information with Expanded View (EV) Figures and Tables that are collapsible/expandable online. A maximum of 5 EV Figures can be typeset. EV Figures should be cited as 'Figure EV1, Figure EV2" etc... in the text and their respective legends should be included in the main text after the legends of regular figures.

- For the figures that you do NOT wish to display as Expanded View figures, they should be bundled together with their legends in a single PDF file called *Appendix*, which should start with a short Table of Content. Appendix figures should be referred to in the main text as: "Appendix Figure S1, Appendix Figure S2" etc.

- Additional Tables/Datasets should be labeled and referred to as Table EV1, Dataset EV1, etc. Legends have to be provided in a separate tab in case of .xls files. Alternatively, the legend can be supplied as a separate text file (README) and zipped together with the Table/Dataset file.

See detailed instructions here:

.

11) The paper explained: EMBO Molecular Medicine articles are accompanied by a summary of the articles to emphasize the major findings in the paper and their medical implications for the non-specialist reader. Please provide a draft summary of your article highlighting

- the medical issue you are addressing,

- the results obtained and

- their clinical impact.

This may be edited to ensure that readers understand the significance and context of the research. Please refer to any of our published articles for an example.

12) For more information: There is space at the end of each article to list relevant web links for further consultation by our readers. Could you identify some relevant ones and provide such information as well? Some examples are patient associations, relevant databases, OMIM/proteins/genes links, author's websites, etc...

13) Author contributions: You will be asked to provide CRediT (Contributor Role Taxonomy) terms in the submission system. These replace a narrative author contribution section in the manuscript.

14) A Conflict of Interest statement should be provided in the main text.

15) Every published paper now includes a 'Synopsis' to further enhance discoverability. Synopses are displayed on the journal webpage and are freely accessible to all readers. They include a short stand first (maximum of 300 characters, including space) as well as 2-5 one-sentences bullet points that summarizes the paper. Please write the bullet points to summarize the key NEW findings. They should be designed to be complementary to the abstract - i.e. not repeat the same text. We encourage inclusion of key acronyms and quantitative information (maximum of 30 words / bullet point). Please use the passive voice. Please attach these in a separate file or send them by email, we will incorporate them accordingly.

Please also suggest a striking image or visual abstract to illustrate your article as a PNG file 550 px wide x 300-600 px high.

EMBO Molecular Medicine has a "scooping protection" policy, whereby similar findings that are published by others during review or revision are not a criterion for rejection. Should you decide to submit a revised version, I do ask that you get in touch after three months if you have not completed it, to update us on the status.

Please note: When submitting your revision you will be prompted to enter your funding and payment information. This will allow Wiley to send you a quote for the article processing charge (APC) in case of acceptance. This quote takes into account any reduction or fee waivers that you may be eligible for. Authors do not need to pay any fees before their manuscript is accepted and transferred to the publisher.

EMBO Press participates in many Publish and Read agreements that allow authors to publish Open Access with reduced/no publication charges. Check your eligibility: https://authorservices.wiley.com/author-resources/Journal-Authors/open-access/affiliation-policies-payments/index.html

***** Reviewer's comments *****

Referee #1 (Comments on Novelty/Model System for Author):

they have chosen the right model to test their hypothesis: detection of plasma proteins possibly serving as biomarker for CHD.

Referee #1 (Remarks for Author):

Yin et al describe an interesting study that they identified a set of plasma proteins, which can be used as biomarkers for pregnant women with CHD. The samples were collected from over 100 pregnant women in the first trimester and well matched with control pregnant women without CHD. The proteomic data were carefully analyzed and some results were further validated by WB. The interpretation of data was appropriate. However, the main concern is on the significance of their findings. The dysregulation of these maternal proteins might be a reflection of exacerbation of certain preclinical conditions of these women under pregnancy, a high stressed condition, regardless of the status (with or without CHD). The argument of control pregnant women without these protein dysregulation is invalid: it might be simply that the control cohort did not have these preclinical conditions. Therefore, another set of proteomics data from same cohort before or after pregnancy will be very helpful to rule out this possibility. Of course, it is very difficult to do that.

Referee #2 (Remarks for Author):

This study attempts to address the problems related to the lack of an accurate method for early detection of congenital heart disease. The findings are interesting and include the identification of new potential biomarkers that combined have a high discriminatory capacity. However, there are limitations ice the enthusiasm and recall a proper methodological approach. The two cohorts recruitment is not clearly illustrated. In particular, the first group consists of women in which the diagnosis was done posnatally, therefore this is in principle a retrospective study unless differently indicated. If this is the case, the criteria to select these particular cases should be mentioned to exclude any potential bias. It is unclear if the investigators were blind to the recruitment and whether the results of the first study was known to the researchers leading the second study. Although the identification of an association between modules and protein pattern is attractive, the authors did not perform any assessment of specificity and sensitivity according to the severity of the disease. This is clinically important as women may be left with an incomplete information of the severity of the disease. The study analyzeed the inter group variability. However, it would be important to assess the reproducibility of data within each case. Finally, the authors assessed the ROC analysis without correction for the prevalence of the disease. Their control group is quantitatively similar to the disease group, but the prevalence of CHD is much lower. Therefore, their conclusion is an overestimation of the predictive value. The discussion does not present any element on how this omic approach can be administered and what would be it practicality and cost-benefit.

Referee #3 (Remarks for Author):

The study by Ya-Nun Yin et al. investigates a relevant topic, trying to answer whether a specific protein expression profile can distinguish mothers of children with congenital heart disease (CHD) from healthy controls.
The study design is simple but appropriate to address the research question.
The findings are intriguing, as some of the de-regulated proteins belong to pathways that are known to be involved in the development of congenital anomalies (e.g. focal adhesion; actin cytoskeleton), while on the other hand some detected proteins are novel associations.
The methodology is appropriate; however, some components are described very briefly.
The identification of clusters is interesting, yet the adoption of machine learning appears overstretched, given the patient numbers. This should be discussed and better justified.
Some of the proteins found to be modulated are involved in lipid metabolism. On one hand, this represents an intriguing finding as dysregulation of lipid metabolism has been suggested to be associated with congenital anomalies such as the bicuspid aorta or Down syndrome. On the other hand, these findings might reflect overfitting, as cases present significantly higher lipid values compared with controls in the study cohort.
The study involves mainly (if not almost exclusively) ASD and VSD CHD patients. This should be clearly stated in the first result paragraph and emphasized in the abstract as well as better presented in the discussion.
Abbreviations of CHD types in Table 1 should be spelled in the note to the table.

Point-by-point Responses to the Reviewers' comments

Referee #1 (Comments on Novelty/Model System for Author):

they have chosen the right model to test their hypothesis: detection of plasma proteins possibly serving as biomarker for CHD.

Referee #1 (Remarks for Author):

Yin et al describe an interesting study that they identified a set of plasma proteins, which can be used as biomarkers for pregnant women with CHD. The samples were collected from over 100 pregnant women in the first trimester and well matched with control pregnant women without CHD. The proteomic data were carefully analyzed and some results were further validated by WB. The interpretation of data was appropriate. However, the main concern is on the significance of their findings. The dysregulation of these maternal proteins might be a reflection of exacerbation of certain preclinical conditions of these women under pregnancy, a high stressed condition, regardless of the status (with or without CHD). The argument of control pregnant women without these protein dysregulation is invalid: it might be simply that the control cohort did not have these preclinical conditions. Therefore, another set of proteomics data from same cohort before or after pregnancy will be very helpful to rule out this possibility. Of course, it is very difficult to do that.

Response: We appreciated the reviewer for the constructive comments. In our study, plasma samples of both case and control groups were collected from pregnant women during early pregnancy (10–12 weeks gestation), so we didn't detect the protein profile before pregnancy nor compare the difference before and after pregnancy. Pregnant women recruited in this study were confirmed not having obvious health problem nor severe metabolic disorders. However, there might be certain preclinical conditions

undetected which can be exacerbated by pregnancy thus disturb the protein profile results. As what the reviewer recommended, another set of proteomics data from same cohort before or after pregnancy would be needed in future study to eliminate this possibility. We would like to further study it when suitable samples were available. Following the reviewer's suggestions, we elaborated the exclusion criteria in the Materials and Methods part and discussed the limitation of this study in the Discussion part in our revised manuscript (lines 372-377, page 19; lines 325-329, page 17).

Referee #2 (Remarks for Author):

This study attempts to address the problems related to the lack of an accurate method for early detection of congenital heart disease. The findings are interesting and include the identification of new potential biomarkers that combined have a high discriminatory capacity. However, there are limitations ice the enthusiasm and recall a proper methodological approach. The two cohorts recruitment is not clearly illustrated. In particular, the first group consists of women in which the diagnosis was done posnatally, therefore this is in principle a retrospective study unless differently indicated. If this is the case, the criteria to select these particular cases should be mentioned to exclude any potential bias.

Response: Thank you for your comprehensive and insightful comments. We apologize for not clarifying the samples recruitment for two groups. In our study, the cases enrolled were the individuals confirmed having CHDs after birth who had echocardiographic evidence. Cases of isolated patent ductus arteriosus, patent foramen ovale, bicuspid aortic valve, coronary anomalies, as well as CHD that relate mainly to the vascular system were excluded from the present study. All patients with genetic syndromes or known chromosomal abnormalities (e.g., Down's syndrome, Holt–Oram syndrome, Alagille syndrome, DiGeorge syndrome, William syndrome, and Noonan syndrome) or

family history of CHD in a first - degree relative (parent, sibling, or child) were excluded. Also, cases combined with other non-cardiovascular malformations, tumor or systematic diseases were not recruited. In our revised manuscript, we elaborated the exclusion criteria in the Materials and Methods part (lines 379-385, page 19).

It is unclear if the investigators were blind to the recruitment and whether the results of the first study was known to the researchers leading the second study.

Response: We apologize for not clarifying the process and the blinding of this research. The recruitment, sample collection and the protein profiling of the group 1 from the Obstetrics & Gynecology Hospital of Fudan University and the group 2 from the International Peace Maternity and Child Health Hospital of the China Welfare Institute were conducted concurrently and independently. The investigators who conduct the following analysis and the building of machine leaning model were blind to the recruitment. The content above was supplemented into the revised Materials and Methods part (lines 367-372, 386-387, page 19-20).

Although the identification of an association between modules and protein pattern is attractive, the authors did not perform any assessment of specificity and sensitivity according to the severity of the disease. This is clinically important as women may be left with an incomplete information of the severity of the disease.

Response: We appreciated the reviewer for the constructive comments. Here in the revised manuscript, we calculated the specificity and sensitivity of our model, which were added in revised Fig 5B, 5E and 5H. In our centers, most critical CHD cases were detected during early or mid-gestation by echocardiogram and aborted according to parents will, leaving no chance for genetic study or postnatal phenotype confirmation. As we only recruited cases who had a complete physical examination, echocardiogram and genetic study by neonatologists and pediatric cardiologists after birth, cases studied were mainly mild VSD and ASD, leaving insufficient data to conduct stratified analysis to

assess the specificity and sensitivity according to the severity of the disease, which is certainly important to the clinical settings. This limitation is further elaborated in the revised Discussion part (lines 330-338, page 17).

The study analyzed the inter group variability. However, it would be important to assess the reproducibility of data within each case.

Response: We appreciate the reviewer’s comment. According to the reviewer’s comment, we further assess the reproducibility of data within each case. In this study, we randomly selected 20 samples (10 CHD and 10 control) from 207 samples to analyze the abundance of correlated proteins across the whole measuring range. The results showed that the average correlation value of quantified protein signals between individual replicates in the healthy control group was 0.978, with a range of 0.96-0.99. Similarly, the average correlation value in the CHD group was 0.965, with a range of 0.93-0.99. However, the average correlation value between CHD and healthy control was 0.939 (Figure EV1A). In addition, to further illustrate the good reproducibility of data within each case, we picked 5 well-characterized plasma proteins and found that the quantification was highly reproducible in 103 healthy control and 104 CHD cases (Figure EV1B) (lines 118-127, page 7; lines 812-816, pages 37-38).

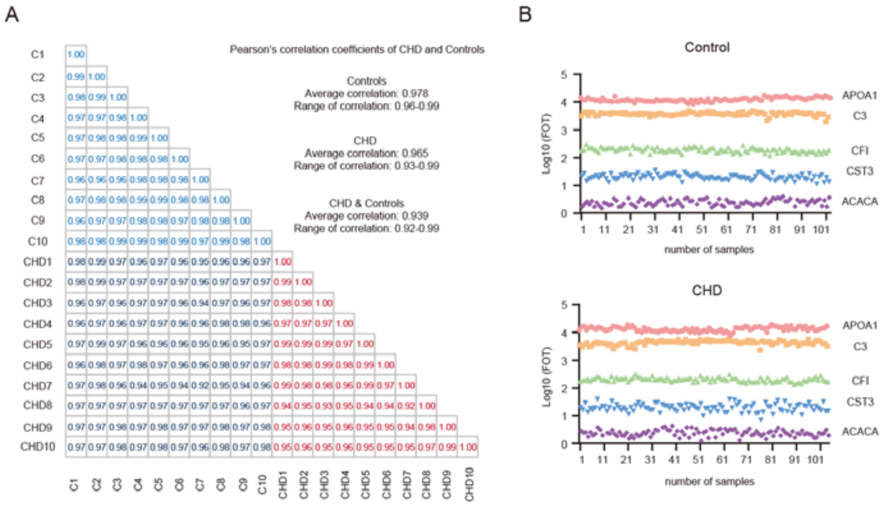

Figure EV1. Reproducibility of plasma data.

A Pearson’s correlation coefficients for replicate proteome profiling of 20 plasma samples

(10 CHD and 10 healthy control samples).

B Reproducibility of the FOT of six proteins in 207 samples. FOT was defined as the iBAQ of a protein divided by the total iBAQ of all identified proteins within a sample.

Finally, the authors assessed the ROC analysis without correction for the prevalence of the disease. Their control group is quantitatively similar to the disease group, but the prevalence of CHD is much lower. Therefore, their conclusion is an overestimation of the predictive value.

Response: We appreciated the reviewer for the constructive comments. We agree with the reviewer and acknowledge that considering the low prevalence of CHD in the general population, the ROC analysis might overestimate the value of this diagnostic approach. We emphasized this limitation in the revised Discussion part and stated that this approach should be applied with caution before verified in larger scale trials (lines 338-342, page 17).

The discussion does not present any element on how this omic approach can be administered and what would be it practicality and cost-benefit.

Response: We appreciated the reviewer for the constructive comments. Only 2 μL plasma from pregnant women at early gestation would allow the administration of this omic approach and guide mom-to-be with high risk of having CHD offspring to receive more intensive follow-up and prepare for prenatal intervention, which certainly helps to the targeted use of medical resources. In the revised manuscript, we elaborated the expectant administration and benefit of this omic approach in the Discussion part (lines 349-353, page 18).

Referee #3 (Remarks for Author):

The study by Ya-Nun Yin et al. investigates a relevant topic, trying to answer whether a specific protein expression profile can distinguish mothers of children with congenital heart disease (CHD) from healthy controls.
The study design is simple but appropriate to address the research question.
The findings are intriguing, as some of the de-regulated proteins belong to pathways that are known to be involved in the development of congenital anomalies (e.g. focal adhesion; actin cytoskeleton), while on the other hand some detected proteins are novel associations.
The methodology is appropriate; however, some components are described very briefly.

Response: We apologize for not clarifying the methodology section. In the revised manuscript, we have expanded the description of the methodology. (lines 441-454, pages 22-23).

The identification of clusters is interesting, yet the adoption of machine learning appears overstretched, given the patient numbers. This should be discussed and better justified.

Response: Thank you for the critical comments. We agree with the reviewer that with this sample size, the conclusion should be drawn with caution and the future administration of this omic approach should be conducted carefully. To reflect the above, we have re-written the related sentences in the Results and Discussion. (lines 338-342, page 17; lines 265 and 270, page 14).

Some of the proteins found to be modulated are involved in lipid metabolism. On one hand, this represents an intriguing finding as dysregulation of lipid metabolism has been suggested to be associated with congenital anomalies such as the bicuspid aorta or Down syndrome. On the other hand, these findings might

reflect overfitting, as cases present significantly higher lipid values compared with controls in the study cohort.

Response: We appreciate the important comment from the reviewer. We agree with the reviewer and acknowledge that the possibility of overfitting considering the proteins involved in lipid metabolism. Further studies comparing the lipid composition and proteins involved in lipid metabolism between case and control groups in larger scale and revealing the underlying mechanism would help to understand the role of lipid metabolism in the occurrence of CHD. This limitation is stated in the Discussion part in our revised manuscript. (lines 342-347, pages 17-18).

The study involves mainly (if not almost exclusively) ASD and VSD CHD patients. This should be clearly stated in the first result paragraph and emphasized in the abstract as well as better presented in the discussion.

Response: We appreciated the reviewer for the constructive comments. In the revised manuscript, we have stated the composition of CHD phenotypes in the first result paragraph and emphasized in the abstract as well as the discussion according to the reviewer's comments. (lines 26-28, page 3; lines 101-102, page 6; lines 333-337, page 17).

Abbreviations of CHD types in Table 1 should be spelled in the note to the table.

Response: We apologize for not writing the abbreviations for the CHD type clearly. In the revised manuscript, we have added the abbreviations of the CHD types in the note to the table, as follows:

VSD: Ventricular septal defect

ASD: Atrial septal defect

PLSVC: Persistent left superior vena cava

PS: Pulmonary stenosis

LVOTO: Left ventricular outflow track obstruction

AVSD: Atrioventricular septal defect

AS: Aortic stenosis

TR: Tricuspid regurgitation

PTA: Persistent truncus arteriosus

TOF: Tetralogy of Fallot

TGA: Transposition of the great arteries

RVOTO: Right ventricular outflow tract obstruction

13th Sep 2023

Dear Dr. Zhao,

Thank you for the submission of your revised manuscript to EMBO Molecular Medicine. I am pleased to inform you that we will be able to accept your manuscript pending the following final amendments:

1) Authors: We note that you currently have 6 first authors and together with you, a total of 4 co-corresponding authors. Is that correct? Do you confirm equal contribution of these authors, able to take full responsibility for the paper and its content? While there is no limit per se to the number of first and co-corresponding authors, 6 first and 4 co-corresponding authors is rare, and may not reflect as intended to the community.
2) In the main manuscript file, please do the following:
- Correct/answer the track changes suggested by our data editors by working from the attached document.
- In M&M, please include statement that the informed consent was obtained from all human subjects and in addition to the principles set out in the WMA Declaration of Helsinki that the experiments conformed to the Department of Health and Human Services Belmont Report.
- Please rename "Conflict of Interest" to "Disclosure Statement & Competing Interests". We updated our journal's competing interests policy in January 2022 and request authors to consider both actual and perceived competing interests. Please review the policy https://www.embopress.org/competing-interests and update your competing interests if necessary.
- Data availability: Please be aware that all deposited data should be freely accessible upon publication. Please use the following format to report the accession number of your data:

The datasets produced in this study are available in the following databases:
[data type]: [full name of the resource] [accession number/identifier] ([doi or URL or identifiers.org/DATABASE:ACCESSION])

Please check "Author Guidelines" for more information.
https://www.embopress.org/page/journal/17574684/authorguide#availabilityofpublishedmaterial
3) Tables: Please rename Tables EV1, EV2, EV3, EV4, EV5 and EV9 to Dataset EV1-6. Tables EV6, EV7, EV8 and EV10 should be updated to Tables EV1-4. Please also update callouts of all tables in the main manuscript text.
4) Synopsis:
- Synopsis text: Please provide a short standfirst (maximum of 300 characters, including space), limit the bullet points to max. 5 and upload it as a separate .doc file. Please write the bullet points to summarise the key NEW findings. They should be designed to be complementary to the abstract - i.e. not repeat the same text. We encourage inclusion of key acronyms and quantitative information (maximum of 30 words / bullet point). Please use the passive voice.
- Synopsis image: Please resize the image to 550 px-wide x (250-400)-px high and upload it as a high-resolution jpeg file.
- Please check your synopsis text and image before submission with your revised manuscript. Please be aware that in the proof stage minor corrections only are allowed (e.g., typos).
5) For more information: This space should be used to list relevant web links for further consultation by our readers. Could you identify some relevant ones and provide such information as well? Some examples are patient associations, relevant databases, OMIM/proteins/genes links, author's websites, etc...
6) As part of the EMBO Publications transparent editorial process initiative (see our Editorial at http://embomolmed.embopress.org/content/2/9/329), EMBO Molecular Medicine will publish online a Review Process File (RPF) to accompany accepted manuscripts. This file will be published in conjunction with your paper and will include the anonymous referee reports, your point-by-point response and all pertinent correspondence relating to the manuscript. Let us know whether you agree with the publication of the RPF and as here, if you want to remove or not any figures from it prior to publication. Please note that the Authors checklist will be published at the end of the RPF.
7) Please provide a point-by-point letter INCLUDING my comments as well as the reviewer's reports and your detailed responses (as Word file).

I look forward to reading a new revised version of your manuscript as soon as possible.

Yours sincerely,

Zeljko Durdevic

Zeljko Durdevic
Editor
EMBO Molecular Medicine

*** Instructions to submit your revised manuscript ***

*** PLEASE NOTE *** As part of the EMBO Publications transparent editorial process initiative (see our Editorial at https://www.embopress.org/doi/pdf/10.1002/emmm.201000094), EMBO Molecular Medicine will publish online a Review Process File to accompany accepted manuscripts.

In the event of acceptance, this file will be published in conjunction with your paper and will include the anonymous referee reports, your point-by-point response and all pertinent correspondence relating to the manuscript. If you do NOT want this file to be published, please inform the editorial office at contact@embomolmed.org.

When submitting your revised manuscript, please include:

1) a .docx formatted version of the manuscript text (including Figure legends and tables)

2) Separate figure files*

3) supplemental information as Expanded View and/or Appendix. Please carefully check the authors guidelines for formatting Expanded view and Appendix figures and tables at https://www.embopress.org/page/journal/17574684/authorguide#expandedview

4) a letter INCLUDING the reviewer's reports and your detailed responses to their comments (as Word file).

5) The paper explained: EMBO Molecular Medicine articles are accompanied by a summary of the articles to emphasize the major findings in the paper and their medical implications for the non-specialist reader. Please provide a draft summary of your article highlighting
- the medical issue you are addressing,
- the results obtained and
- their clinical impact.
This may be edited to ensure that readers understand the significance and context of the research.
Please refer to any of our published articles for an example.

6) For more information: There is space at the end of each article to list relevant web links for further consultation by our readers. Could you identify some relevant ones and provide such information as well? Some examples are patient associations, relevant databases, OMIM/proteins/genes links, author's websites, etc...

7) Author contributions: the contribution of every author must be detailed in a separate section.

8) EMBO Molecular Medicine now requires a complete author checklist (https://www.embopress.org/page/journal/17574684/authorguide) to be submitted with all revised manuscripts. Please use the checklist as guideline for the sort of information we need WITHIN the manuscript. The checklist should only be filled with page numbers were the information can be found. This is particularly important for animal reporting, antibody dilutions (missing) and exact values and n that should be indicted instead of a range.

9) Every published paper now includes a 'Synopsis' to further enhance discoverability. Synopses are displayed on the journal webpage and are freely accessible to all readers. They include a short stand first (maximum of 300 characters, including space) as well as 2-5 one sentence bullet points that summarise the paper. Please write the bullet points to summarise the key NEW findings. They should be designed to be complementary to the abstract - i.e. not repeat the same text. We encourage inclusion of key acronyms and quantitative information (maximum of 30 words / bullet point). Please use the passive voice. Please attach these in a separate file or send them by email, we will incorporate them accordingly.

You are also welcome to suggest a striking image or visual abstract to illustrate your article. If you do please provide a jpeg file 550 px-wide x 400-px high.

10) A Conflict of Interest statement should be provided in the main text

11) Please note that we now mandate that all corresponding authors list an ORCID digital identifier. This takes <90 seconds to

complete. We encourage all authors to supply an ORCID identifier, which will be linked to their name for unambiguous name identification.

Currently, our records indicate that the ORCID for your account is 0000-0001-9861-0330.

Please click the link below to modify this ORCID:
Link Not Available

12) The system will prompt you to fill in your funding and payment information. This will allow Wiley to send you a quote for the article processing charge (APC) in case of acceptance. This quote takes into account any reduction or fee waivers that you may be eligible for. Authors do not need to pay any fees before their manuscript is accepted and transferred to our publisher.

*Additional important information regarding Figures

Each figure should be given in a separate file and should have the following resolution:
Graphs 800-1,200 DPI
Photos 400-800 DPI
Colour (only CMYK) 300-400 DPI"

Figures are not edited by the production team. All lettering should be the same size and style; figure panels should be indicated by capital letters (A, B, C etc). Gridlines are not allowed except for log plots. Figures should be numbered in the order of their appearance in the text with Arabic numerals. Each Figure must have a separate legend and a caption is needed for each panel.

*Additional important information regarding figures and illustrations can be found at https://bit.ly/EMBOPressFigurePreparationGuideline. See also figure legend preparation guidelines: https://www.embopress.org/page/journal/17574684/authorguide#figureformat

The system will prompt you to fill in your funding and payment information. This will allow Wiley to send you a quote for the article processing charge (APC) in case of acceptance. This quote takes into account any reduction or fee waivers that you may be eligible for. Authors do not need to pay any fees before their manuscript is accepted and transferred to our publisher.

***** Reviewer's comments *****

Referee #1 (Remarks for Author):

the authors have addressed my concerns at some level. I had no further concerns.

Referee #2 (Remarks for Author):

The authors have responded my questions

Referee #3 (Remarks for Author):

The authors have satisfactorily addressed my comments and have overall strengthened their manuscript through this round of revision.

The authors addressed the remaining editorial issues.

27th Sep 2023

Dear Dr. Zhao,

We are pleased to inform you that your manuscript is accepted for publication and is now being sent to our publisher to be included in the next available issue of EMBO Molecular Medicine.

Please read below for additional IMPORTANT information regarding your article, its publication and the production process.

Congratulations on your interesting work,

Zeljko Durdevic

Zeljko Durdevic
Editor
EMBO Molecular Medicine

Follow us on Twitter @EmboMolMed
Sign up for eTOCs at embopress.org/alertsfeeds

*** *** *** IMPORTANT INFORMATION *** *** ***

SPEED OF PUBLICATION
The journal aims for rapid publication of papers, using using the advance online publication "Early View" to expedite the process: A properly copy-edited and formatted version will be published as "Early View" after the proofs have been corrected. Please help the Editors and publisher avoid delays by providing e-mail address(es), telephone and fax numbers at which author(s) can be contacted.

Should you be planning a Press Release on your article, please get in contact with embomolmed@wiley.com as early as possible, in order to coordinate publication and release dates.

LICENSE AND PAYMENT:

All articles published in EMBO Molecular Medicine are fully open access: immediately and freely available to read, download and share.

EMBO Molecular Medicine charges an article processing charge (APC) to cover the publication costs. You, as the corresponding author for this manuscript, should have already received a quote with the article processing fee separately. Please let us know in case this quote has not been received.

Once your article is at Wiley for editorial production you will receive an email from Wiley's Author Services system, which will ask you to log in and will present you with the publication license form for completion. Within the same system the publication fee can be paid by credit card, an invoice, pro forma invoice or purchase order can be requested.

Payment of the publication charge and the signed Open Access Agreement form must be received before the article can be published online.

PROOFS

You will receive the proofs by e-mail approximately 2 weeks after all relevant files have been sent o our Production Office. Please return them within 48 hours and if there should be any problems, please contact the production office at embopressproduction@wiley.com.

Please inform us if there is likely to be any difficulty in reaching you at the above address at that time. Failure to meet our

deadlines may result in a delay of publication.

All further communications concerning your paper proofs should quote reference number EMM-2023-17745-V3 and be directed to the production office at embopressproduction@wiley.com.

Thank you,

Zeljko Durdevic
Editor
EMBO Molecular Medicine

EMBO Press Author Checklist

Corresponding Author Name: Jian-yuan Zhao	
Journal Submitted to: EMBO Molecular Medicine	
Manuscript Number: EMM-2023-17745-V2	

USEFUL LINKS FOR COMPLETING THIS FORM
The EMBO Journal - Author Guidelines
EMBO Reports - Author Guidelines
Molecular Systems Biology - Author Guidelines
EMBO Molecular Medicine - Author Guidelines

Reporting Checklist for Life Science Articles (updated January
This checklist is adapted from Materials Design Analysis Reporting (MDAR) Checklist for Authors. MDAR establishes a minimum set of requirements in transparent reporting in the life sciences (see Statement of Task: 10.31222/osf.io/9sm4x). Please follow the journal's guidelines in preparing your
Please note that a copy of this checklist will be published alongside your article.

Abridged guidelines for figures
1. Data
The data shown in figures should satisfy the following conditions:
- the data were obtained and processed according to the field's best practice and are presented to reflect the results of the experiments in an accurate and unbiased manner.
- ideally, figure panels should include only measurements that are directly comparable to each other and obtained with the same assay.
- plots include clearly labeled error bars for independent experiments and sample sizes. Unless justified, error bars should not be shown for technical
- if n<5, the individual data points from each experiment should be plotted. Any statistical test employed should be justified.
- Source Data should be included to report the data underlying figures according to the guidelines set out in the authorship guidelines on Data

2. Captions
Each figure caption should contain the following information, for each panel where they are relevant:
- a specification of the experimental system investigated (eg cell line, species name).
- the assay(s) and method(s) used to carry out the reported observations and measurements.
- an explicit mention of the biological and chemical entity(ies) that are being measured.
- an explicit mention of the biological and chemical entity(ies) that are altered/varied/perturbed in a controlled manner.
- the exact sample size (n) for each experimental group/condition, given as a number, not a range;
- a description of the sample collection allowing the reader to understand whether the samples represent technical or biological replicates (including how many animals, litters, cultures, etc.).
- a statement of how many times the experiment shown was independently replicated in the laboratory.
- definitions of statistical methods and measures:
  - common tests, such as t-test (please specify whether paired vs. unpaired), simple χ2 tests, Wilcoxon and Mann-Whitney tests, can be unambiguously identified by name only, but more complex techniques should be described in the methods section;
  - are tests one-sided or two-sided?
  - are there adjustments for multiple comparisons?
  - exact statistical test results, e.g., P values = x but not P values < x;
  - definition of 'center values' as median or average;
  - definition of error bars as s.d. or s.e.m.

Please complete ALL of the questions below.
Select "Not Applicable" only when the requested information is not relevant for your study.

Materials

Newly Created Materials	Information included in the manuscript?	In which section is the information available? (Reagents and Tools Table, Materials and Methods, Figures, Data Availability Section)
New materials and reagents need to be available; do any restrictions apply?	Not Applicable	

Antibodies	Information included in the manuscript?	In which section is the information available? (Reagents and Tools Table, Materials and Methods, Figures, Data Availability Section)
For **antibodies** provide the following information:<br>- Commercial antibodies: RRID (if possible) or supplier name, catalogue number and or/clone number<br>- Non-commercial: RRID or citation	Yes	Materials and Methods

DNA and RNA sequences	Information included in the manuscript?	In which section is the information available? (Reagents and Tools Table, Materials and Methods, Figures, Data Availability Section)
**Short novel DNA or RNA including primers, probes**: provide the sequences.	Not Applicable	

Cell materials	Information included in the manuscript?	In which section is the information available? (Reagents and Tools Table, Materials and Methods, Figures, Data Availability Section)
**Cell lines:** Provide species information, strain. Provide accession number in repository **OR** supplier name, catalog number, clone number, and/**OR** RRID.	Not Applicable	
**Primary cultures:** Provide species, strain, sex of origin, genetic modification status.	Not Applicable	
Report if the cell lines were recently **authenticated** (e.g., by STR profiling) and tested for mycoplasma contamination.	Not Applicable	

Experimental animals	Information included in the manuscript?	In which section is the information available? (Reagents and Tools Table, Materials and Methods, Figures, Data Availability Section)
**Laboratory animals or Model organisms:** Provide species, strain, sex, age, genetic modification status. Provide accession number in repository **OR** supplier name, catalog number, clone number, **OR** RRID.	Not Applicable	
**Animal observed in or captured from the field:** Provide species, sex, and age where possible.	Not Applicable	
Please detail **housing and husbandry conditions**.	Not Applicable	

Plants and microbes	Information included in the manuscript?	In which section is the information available? (Reagents and Tools Table, Materials and Methods, Figures, Data Availability Section)
**Plants:** provide species and strain, ecotype and cultivar where relevant, unique accession number if available, and source (including location for collected wild specimens).	Not Applicable	
**Microbes:** provide species and strain, unique accession number if available, and source.	Not Applicable	

Human research participants	Information included in the manuscript?	In which section is the information available? (Reagents and Tools Table, Materials and Methods, Figures, Data Availability Section)
If collected and within the bounds of privacy constraints report on age, sex and gender or ethnicity for all study participants.	Yes	Materials and Methods

Core facilities	Information included in the manuscript?	In which section is the information available? (Reagents and Tools Table, Materials and Methods, Figures, Data Availability Section)
If your work benefited from core facilities, was their service mentioned in the acknowledgments section?	Not Applicable	

Design

Study protocol	Information included in the manuscript?	In which section is the information available? (Reagents and Tools Table, Materials and Methods, Figures, Data Availability Section)
If study protocol has been **pre-registered, provide DOI in the manuscript**. For clinical trials, provide the trial registration number **OR** cite DOI.	Not Applicable	
Report the **clinical trial registration number** (at ClinicalTrials.gov or equivalent), where applicable.	Not Applicable	

Laboratory protocol	Information included in the manuscript?	In which section is the information available? (Reagents and Tools Table, Materials and Methods, Figures, Data Availability Section)
Provide DOI OR other citation details if **external detailed step-by-step protocols** are available.	Yes	Materials and Methods

Experimental study design and statistics	Information included in the manuscript?	In which section is the information available? (Reagents and Tools Table, Materials and Methods, Figures, Data Availability Section)
Include a statement about **sample size** estimate even if no statistical methods were used.	Not Applicable	
Were any steps taken to minimize the effects of subjective bias when allocating animals/samples to treatment (e.g. **randomization procedure**)? If yes, have they been described?	Yes	Materials and Methods
Include a statement about **blinding** even if no blinding was done.	Yes	Materials and Methods
Describe **inclusion/exclusion criteria** if samples or animals were excluded from the analysis. Were the criteria pre-established?<br><br>If sample or data points were omitted from analysis, report if this was due to attrition or intentional exclusion and provide justification.	Yes	Materials and Methods
For every figure, are **statistical tests** justified as appropriate? Do the data meet the assumptions of the tests (e.g., normal distribution)? Describe any methods used to assess it. Is there an estimate of variation within each group of data? Is the variance similar between the groups that are being statistically compared?	Yes	Materials and Methods

Sample definition and in-laboratory replication	Information included in the manuscript?	In which section is the information available? (Reagents and Tools Table, Materials and Methods, Figures, Data Availability Section)
In the figure legends: state number of times the experiment was **replicated** in laboratory.	Not Applicable	
In the figure legends: define whether data describe **technical or biological replicates**.	Not Applicable	

Ethics

Ethics	Information included in the manuscript?	In which section is the information available? (Reagents and Tools Table, Materials and Methods, Figures, Data Availability Section)
Studies involving **human participants**: State details of **authority granting ethics approval** (IRB or equivalent committee(s), provide reference number for approval.	Yes	Materials and Methods
Studies involving **human participants**: Include a statement confirming that **informed consent** was obtained from all subjects and that the experiments conformed to the principles set out in the WMA Declaration of Helsinki and the Department of Health and Human Services Belmont Report.	Yes	Materials and Methods
Studies involving **human participants: For** publication of **patient photos**, include a statement confirming that consent to publish was obtained.	Not Applicable	
Studies involving experimental **animals**: State details of **authority granting ethics approval** (IRB or equivalent committee(s), provide reference number for approval. Include a statement of compliance with ethical regulations.	Not Applicable	
Studies involving **specimen and field samples:** State if relevant **permits** obtained, provide details of authority approving study; if none were required, explain why.	Yes	Materials and Methods

Dual Use Research of Concern (DURC)	Information included in the manuscript?	In which section is the information available? (Reagents and Tools Table, Materials and Methods, Figures, Data Availability Section)
Could your study fall under dual use research restrictions? Please check biosecurity documents and list of **select agents and toxins** (CDC): https://www.selectagents.gov/sat/list.htm	Not Applicable	
If you used a select agent, is the security level of the lab appropriate and reported in the manuscript?	Not Applicable	
If a study is subject to dual use research of concern regulations, is the name of the **authority granting approval and reference number** for the regulatory approval provided in the manuscript?	Not Applicable	

Reporting

The MDAR framework recommends adoption of discipline-specific guidelines, established and endorsed through community initiatives. Journals have their own policy about requiring specific guidelines and recommendations to complement MDAR.

Adherence to community standards	Information included in the manuscript?	In which section is the information available? (Reagents and Tools Table, Materials and Methods, Figures, Data Availability Section)
State if relevant guidelines or checklists (e.g., **ICMJE, MIBBI, ARRIVE, PRISMA**) have been followed or provided.	Not Applicable	
For **tumor marker prognostic studies,** we recommend that you follow the **REMARK** reporting guidelines (see link list at top right). See author guidelines, under 'Reporting Guidelines'. Please confirm you have followed these guidelines.	Not Applicable	
For **phase II and III randomized controlled trials**, please refer to the **CONSORT** flow diagram (see link list at top right) and submit the CONSORT checklist (see link list at top right) with your submission. See author guidelines, under 'Reporting Guidelines'. Please confirm you have submitted this list.	Not Applicable	

Data Availability

Data availability	Information included in the manuscript?	In which section is the information available? (Reagents and Tools Table, Materials and Methods, Figures, Data Availability Section)
Have **primary datasets** been deposited according to the journal's guidelines (see 'Data Deposition' section) and the respective accession numbers provided in the Data Availability Section?	Yes	Data Availability Section
Were **human clinical and genomic datasets** deposited in a public access-controlled repository in accordance to ethical obligations to the patients and to the applicable consent agreement?	Not Applicable	
Are **computational models** that are central and integral to a study available without restrictions in a machine-readable form? Were the relevant accession numbers or links provided?	Not Applicable	
If publicly available data were reused, provide the respective **data citations in the reference list**.	Not Applicable	
